# Myelin dysfunction drives amyloid-β deposition in models of Alzheimer’s disease

**DOI:** 10.1038/s41586-023-06120-6

**Published:** 2023-05-31

**Authors:** Constanze Depp, Ting Sun, Andrew Octavian Sasmita, Lena Spieth, Stefan A. Berghoff, Taisiia Nazarenko, Katharina Overhoff, Agnes A. Steixner-Kumar, Swati Subramanian, Sahab Arinrad, Torben Ruhwedel, Wiebke Möbius, Sandra Göbbels, Gesine Saher, Hauke B. Werner, Alkmini Damkou, Silvia Zampar, Oliver Wirths, Maik Thalmann, Mikael Simons, Takashi Saito, Takaomi Saido, Dilja Krueger-Burg, Riki Kawaguchi, Michael Willem, Christian Haass, Daniel Geschwind, Hannelore Ehrenreich, Ruth Stassart, Klaus-Armin Nave

**Affiliations:** 1 Department of Neurogenetics, Max Planck Institute for Multidisciplinary Sciences, Göttingen, Germany; 2 Institute of Neuronal Cell Biology, Technical University Munich, Munich, Germany; 3 German Center for Neurodegenerative Diseases, Munich, Germany; 4 Clinical Neuroscience, Max Planck Institute for Multidisciplinary Sciences, Göttingen, Germany; 5 Department of Psychiatry and Psychotherapy, University Medical Center, Georg-August University, Göttingen, Germany; 6 Department of German Philology, Georg-August University, Göttingen, Germany; 7 Munich Cluster of Systems Neurology (SyNergy), Munich, Germany; 8 Department of Neurocognitive Science, Institute of Brain Science, Nagoya City University Graduate School of Medical Sciences, Nagoya, Aichi, Japan; 9 Laboratory for Proteolytic Neuroscience, RIKEN Center for Brain Science, Wako, Saitama, Japan; 10 Department of Molecular Neurobiology, Max Planck Institute for Multidisciplinary Sciences, Göttingen, Germany; 11 Program in Neurogenetics, Department of Neurology, David Geffen School of Medicine, University of California Los Angeles, Los Angeles, CA USA; 12 Metabolic Biochemistry, Biomedical Center (BMC), Faculty of Medicine, Ludwig-Maximilians University of Munich, Munich, Germany; 13 Paul-Flechsig-Institute of Neuropathology, University Clinic Leipzig, Leipzig, Germany

**Keywords:** Oligodendrocyte, Alzheimer's disease

## Abstract

The incidence of Alzheimer’s disease (AD), the leading cause of dementia, increases rapidly with age, but why age constitutes the main risk factor is still poorly understood. Brain ageing affects oligodendrocytes and the structural integrity of myelin sheaths^1^, the latter of which is associated with secondary neuroinflammation^2,3^. As oligodendrocytes support axonal energy metabolism and neuronal health^4–7^, we hypothesized that loss of myelin integrity could be an upstream risk factor for neuronal amyloid-β (Aβ) deposition, the central neuropathological hallmark of AD. Here we identify genetic pathways of myelin dysfunction and demyelinating injuries as potent drivers of amyloid deposition in mouse models of AD. Mechanistically, myelin dysfunction causes the accumulation of the Aβ-producing machinery within axonal swellings and increases the cleavage of cortical amyloid precursor protein. Suprisingly, AD mice with dysfunctional myelin lack plaque-corralling microglia despite an overall increase in their numbers. Bulk and single-cell transcriptomics of AD mouse models with myelin defects show that there is a concomitant induction of highly similar but distinct disease-associated microglia signatures specific to myelin damage and amyloid plaques, respectively. Despite successful induction, amyloid disease-associated microglia (DAM) that usually clear amyloid plaques are apparently distracted to nearby myelin damage. Our data suggest a working model whereby age-dependent structural defects of myelin promote Aβ plaque formation directly and indirectly and are therefore an upstream AD risk factor. Improving oligodendrocyte health and myelin integrity could be a promising target to delay development and slow progression of AD.

## Abstract (web summary)

Mouse models show that myelin dysfunction and associated inflammation increase with age, which can promote amyloid-β deposition and therefore risk of developing Alzheimer’s disease.

## Main

The pathology of AD is characterized by the deposition of Aβ plaques and neurofibrillary tangles primarily in the neocortex and hippocampus. According to the amyloid hypothesis of AD, the build-up of Aβ initiates a cascade of harmful events that lead to neuronal dysfunction. More than lifestyle choices and genetic predisposition, old age is the primary risk factor for AD development, but exactly how brain ageing is linked to amyloid deposition is unclear. Myelin, a spirally wrapped and compacted glial membrane, enhances axonal conduction speed, and its non-compacted regions allow oligodendrocytes to provide metabolic support to the neuronal compartment^7^. The specific cellular architecture of myelin makes protein and lipid turnover challenging and slow^8,9^. This, together with the long lifetime of oligodendrocytes^10^, might explain the structural deterioration of myelin with age^1^. We speculated that the breakdown of myelin integrity in the ageing brain acts as a driving force for Aβ deposition. We tested this hypothesis in mouse models of amyloidosis, in which we used genetic and pharmacological manipulation to introduce various degrees of myelin dysfunction.

## Myelin decline in patients with AD

**Fig. 1:**
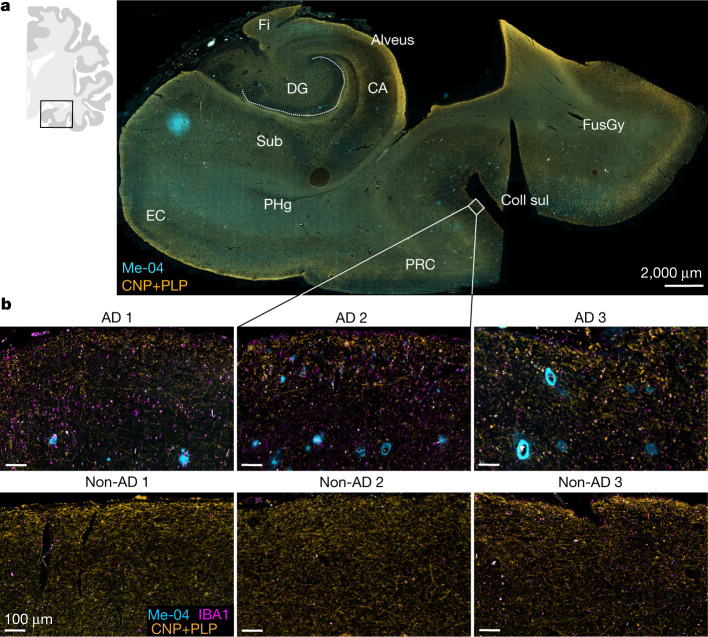
Myelin damage in patients with AD. Fluorescent immunolabelling of CNP and PLP (myelinated fibres) and IBA1 (microglia) and Me-04 staining (Aβ plaques) in the medial temporal lobe of patients with AD and in unaffected individuals (non-AD). **a**, Annotated overview image of the medial temporal lobe of a patient with AD indicating the location of magnified images. **b**, Magnified images of the upper cortical layers in the transentorhinal cortex in patients with AD and in unaffected individuals (*n* = 3 per group). CA, cornu ammonis; Coll sul: collateral sulcus; DG, dentate gyrus; EC, entorhinal cortex; Fi, fimbria; FusGy, fusiform gyrus; PHg, parahippocampal gyrus; PRC, perirhinal cortex; Sub, subiculum.

**Extended Data Fig. 1:**
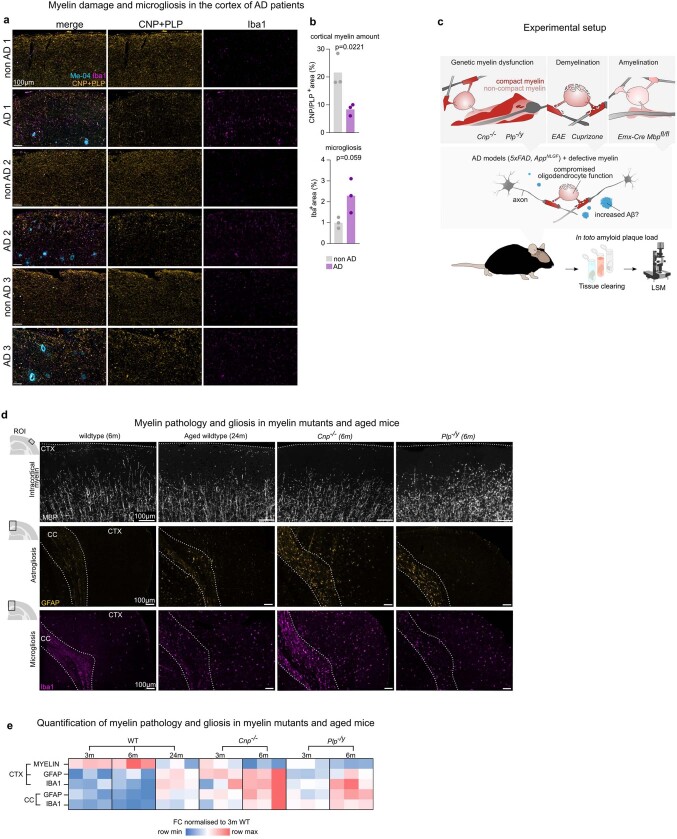
Cortical myelin levels and gliosis in AD patients, aged wildtype mice and myelin mutants. Continuation of Fig. 1. (**a**) Merge and single channel images showing microglia (stained with Iba1) and myelin levels (stained with PLP/CNP) as shown in Fig. 1. (**b**) Quantification of microgliosis and myelin levels in AD patients and non-AD controls as percentage area covered in the cortical areas examined. Bars represent means; dots represent individual patients/biological replicate/n (n = 3 per group). Statistical analysis: two-sided, unpaired Student’s t-test (p-value is given in graph). (**c**) Experimental setup to study the effect of myelin dysfunction on amyloid plaque load in AD mouse models. Models of genetic myelin dysfunction, acute demyelination and genetic amyelination were combined with the *5xFAD* and *App*^*NLGF*^ model of AD and effects on amyloid deposition were investigated *in toto* by tissue clearing and light sheet microscopy (LSM). (**d**) Myelin defects drive a premature ageing phenotype in mice. Panels show microscopic images of immunolabellings against MBP (Myelin), GFAP (Astrogliosis), and Iba1 (Microgliosis) in 6-month-old wildtype, 24-month-old wildtype, 6-month-old myelin mutant (*Cnp*^*−/−*^ and *Plp*^*−/y*^) mice. Regions of interest (ROI) analysed are indicated on the left. Dashed lines in the upper panel mark the pial surface above the cortex (CTX). Dashed lines in lower panels outline the corpus callosum (CC). (**e**) Heatmap representation of the quantification of myelin pathology and gliosis immunostainings as shown in (c) in myelin mutants (*Cnp*^*−/−*^ and *Plp*^*−/y*^) and wildtype mice at 3-months (3m), 6-months (6m) and 24-months (24m). The percentage area covered was quantified in the indicated ROIs and is presented as fold-change (FC) with respect to the 3-month-old data set. Each heatmap tile represents a single animal/biological replicate/n (n = 3 per group) and is colour-coded according to the row minimum-maximum lookup table shown below. Statistical analysis and raw values are given in Supplementary Fig. 2. Source data

Macroscopic-scale brain imaging studies have suggested that cortical myelin damage occurs in the preclinical phase of AD^11–14^. Microscopy-based evidence of this damage, however, remains scarce. We therefore used immunofluorescence to analyse intracortical myelin integrity in a small group of patients with AD, focusing on the trans-entorhinal area (Fig. 1 and Extended Data Fig. 1a,b). We observed a decline in intracortical myelin density in autopsy samples from patients with AD that were not limited to the immediate vicinity of plaques. Sites of myelin loss were also associated with increased numbers of IBA1^+^ microglia (Extended Data Fig. 1a,b). Correlations in human neuropathology cannot determine whether myelin loss is the cause or consequence of neuronal AD pathology such as axonal decay. To clarify this issue, we used two different experimental animal models of AD (5×FAD and *APP*^*NLGF*^; Extended Data Fig. 1c) to investigate whether loss of myelin can act as an upstream driver of amyloidosis. Specifically, we combined the AD mouse models with mice that develop subtle myelin disintegration that is induced using genetic methods (Extended Data Fig. 1c). Mice with loss of function (null mutants) of the myelin architectural proteins CNP and PLP display minor structural myelin defects: lack of CNP causes the collapse of cytosolic channels within the myelin sheath^15^ associated with axonal swellings^16^. PLP-deficient myelin lacks physical stability, impairs axonal energy metabolism^17^ and causes axonopathy at higher age^4^. We first compared normal brain ageing of C57BL/6 wild-type (WT) mice with that of *Cnp*^*−/−*^ mice and *Plp*^*−/y*^ mice on the same background. Immunostaining showed that *Cnp*^*−/−*^ and *Plp*^*−/y*^ mice have progressive gliosis in grey and white matter between the age of 3 and 6 months. The progressive gliosis was qualitatively similar to aged WT mice (24 months old) but more substantial (Extended Data Fig. 1d,e and Supplementary Fig.  2). Similar to patients with AD, the aged WT mice and more so the myelin-defective mutant mice displayed significant reductions in intracortical myelin content, especially in the upper cortical layers (Extended Data Fig. 1d,e).

## Myelin defects exacerbate Aβ deposition

**Fig. 2:**
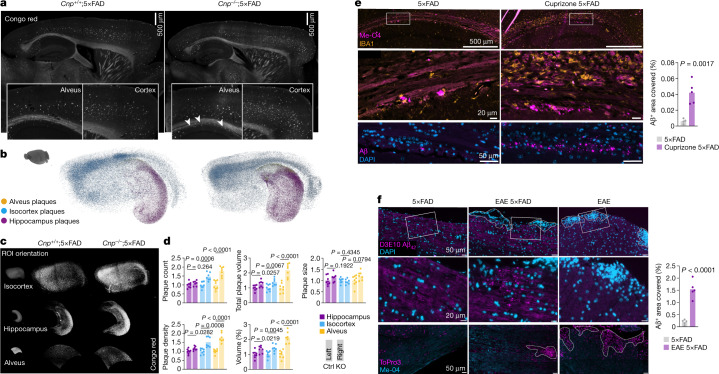
Dysmyelination and demyelination enhance amyloid plaque deposition in 5×FAD mice. **a**–**d**, LSM analysis of 6-month-old *Cnp*^*+/+*^;5xFAD and *Cnp*^*−/−*^;5xFAD mouse brains stained for Congo red. **a**, Representative LSM 2D single planes. Inlays show close-up images of the cortex and alveus. Arrowheads indicate small amyloid deposits in the alveus. **b**, 3D representation of hippocampal, isocortical and alveus plaques represented as coloured centroids. **c**, 3D cropped regions of interest (ROIs) of a representative brain rendered in maximum intensity modus. **d**, 3D quantification of plaque load in the indicated ROIs normalized to the controls. *n* = 8 for control, *n* = 7 for mutant. Ctrl, control; KO, knockout. **e**, Left, 2D immunostaining images of microglia (IBA1) and Aβ plaques with Me-04 (top two rows) or antibody-labelling (bottom row) in the alveus of cuprizone-treated 5×FAD mice (cuprizone 5×FAD) and control animals (5xFAD). Right, quantification of amyloid-positive deposits in the alveus. *n* = 4 for control, *n* = 5 for cuprizone treatment. **f**, Left, 2D immunostaining images of amyloid using antibody labelling (top two rows) or the β-sheet dye Me-04 (bottom row). EAE lesions are indicated by nuclei accumulations (DAPI or ToPro3 labelling) and marked by dashed lines. EAE control animals are shown to rule out nonspecific staining of lesion sites in EAE. Right, quantification of amyloid-positive deposits in the lesion environment. *n* = 5 for control, *n* = 5 for EAE treatment. For **d**–**f**, statistical analysis: two-sided, unpaired Student’s *t*-test (*P* values are indicated in the graphs). Bars represent the means, dots represent biological replicates/mice/*n*. Source data

**Extended Data Fig. 2:**
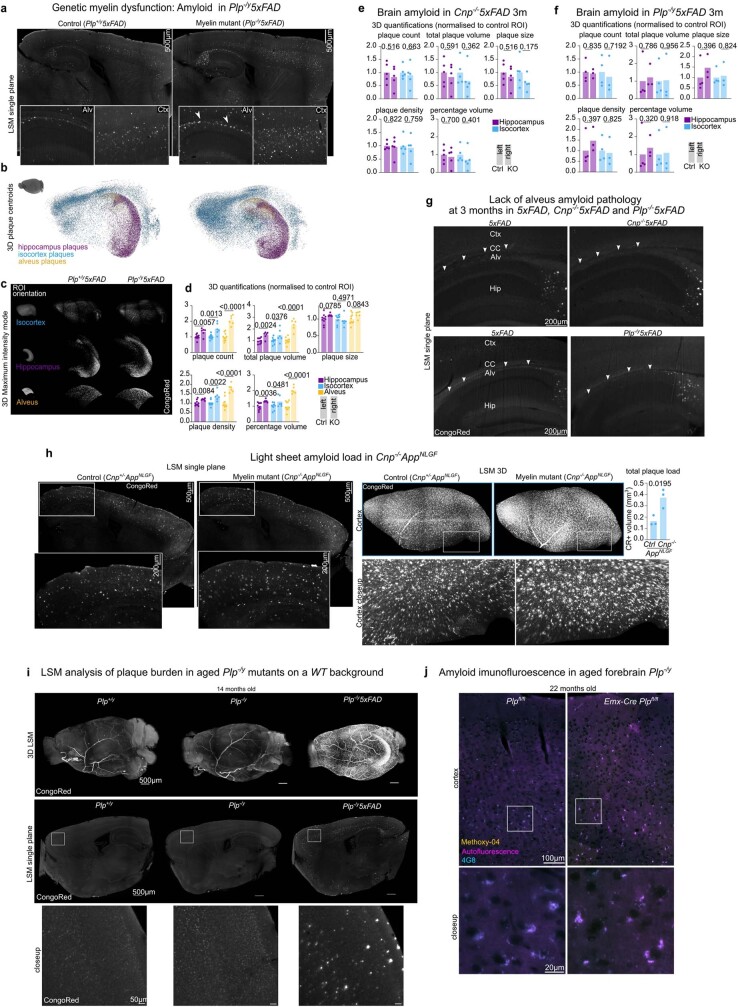
Microscopic analysis of plaque changes induced by genetic dysmyelination at 3 months of age in *5xFAD* mice, 6-month old *App*^*NLGF*^ mice and myelin mutants on a WT background. Extension of Fig. 2. (**a**-**d**) LSM analysis of 6-month-old *Plp*^*+/y*^*5xFAD* and *Plp*^*−/y*^*5xFAD* brains stained for Congo Red. (**a**) Representative LSM 2D single planes Inlays show closeup images of the cortex (Ctx) and alveus (Alv). Arrows indicate small amyloid deposits in the alveus. (**b**) 3D representation of hippocampal, isocortical and alveus plaques represented as coloured centroids. (**c**) 3D cropped regions of interest of brains rendered in maximum intensity modus. (**d**) 3D quantification of plaque load in the three regions of interest normalised to the respective region in control animals. Bars represent means; dots represent biological replicates/mice/n (n = 10 for control, n = 6 for mutant). Statistical analysis: two-sided, unpaired Student’s t-test (p-values are given above bars). (**e**) Quantifications of light sheet microscopic analysis of 3-months-old *Cnp*^*−/−*^*5xFAD* mice. Bars represent means; dots represent biological replicates/mice/n (n = 5 for control, n = 5 for KO). Statistical analysis: two-sided, unpaired Student’s t-test (p-values are given above bars). (**f**) Quantifications of light sheet microscopic analysis of 3-months-old *Plp*^*−/y*^*5xFAD* mice. Bars represent means; dots represent biological replicates/mice/n (n = 4 for control, n = 3 for KO). Statistical analysis: two-sided, unpaired Student’s t-test (p-values are given above bars). (**g**) Light sheet microscopic single plane closeup of the alveus region of Congo red stained brains of 3-month old *5xFAD* control mice, *Cnp*^*−/−*^*5xFAD*, and *Plp*^*−/y*^*5xFAD* myelin mutants. Note the absence of plaques in the alveus at this age. Arrowheads indicate the alveus. CC:Corpus callosum, Alv: Alveus. Ctx: Cortex. Hip: Hippocampus. (**h**) Light sheet microscopic analysis of *Cnp*^*−/−*^*App*^*NLGF*^ brains shows enhanced plaque deposition at 6-month of age when compared to *Cnp*^*+/−*^*App*^*NLGF*^ controls. Left panel shows LSM single plane and a closeup of a cortical region. Right panel shows 3D maximum intensity projection of the cropped isocortical ROI. Plaque burden was quantified using machine-learning-based segmentation of amyloid plaques. Bars represent means; dots represent biological replicates/mice/n (n = 3 per group). Statistical analysis: two-sided, unpaired Student’s t-test (p-value is given in graph). (**i**) Light sheet microscopic analysis of plaque burden in aged (14-months old) *Plp*^*−/y*^ brains on a *WT* background. As a positive control, a 14-months old *5xFAD* brain with ample plaque pathology is shown. In aged *WT* and *Plp*^*−/y*^ only non-specific nuclei or lipofuscin signals can be observed. No plaque-like structures could be identified in the whole brain. The same observations were made in three independent samples/mice for *WT* and *Plp*^*−/y*^. (**j**) Immunofluorescence staining of β-sheet positive plaques (Methoxy-04) and amyloid-β (4G8) in aged (22-months old) forebrain-specific PLP knockout animals (*Emx-Cre Plp*^*fl/fl*^). Note ample lipofuscin accumulation (autofluorescence) colocalising fully with signal in the 4G8 channel. No plaque-like structures could be identified in both groups. The same observations were made for four independent samples/mice per group. Source data

To determine whether such ageing-associated myelin defects can drive amyloid deposition, we crossbred *Cnp*^*−/−*^ and *Plp*^*−/y*^ mutants with 5×FAD mice and analysed plaque burden in the resulting offspring (Extended Data Fig. 1c). For this analysis, we optimized a Congo red in toto plaque staining and clearing protocol based on the iDISCO technique for light sheet microscopy (LSM) (Supplementary Fig. 3) to determine the amyloid plaque load in the entire brain in an unbiased fashion. When compared with 5×FAD mice, both *Cnp*^*−/−*^;5×FAD mice and *Plp*^*−/y*^;5×FAD mice showed marked increases in amyloid plaque load in hippocampal white matter (alveus) and cortex at 6 months of age (Fig. 2a–d and Extended Data Fig. 2a–d). In both mouse models, the effects were strongest in the alveus, a hippocampal white matter tract: here Aβ was deposited in very small aggregates, which indicated an increased formation of amyloid seeds. Increases in plaque load were observed at 6 months but not yet at 3 months of age (Extended Data Fig. 2e,f) when *Cnp*^*−/−*^ mice and *Plp*^*−/y*^ mice exhibited only subtle myelin defects and reactive gliosis (Extended Data Fig. 1d). Moreover, 5×FAD control mice and the myelin mutant mice did not show plaque pathology in the alveus at this age (Extended Data Fig. 2g). To exclude the possibility that these effects are specific to the 5×FAD model and *App* overexpression, we validated our findings in crossbreedings of *Cnp*^*−/−*^ mice and the *APP*^*NLGF*^ knock-in model, in which three mutations in the human gene encoding amyloid precursor protein (*APP*) is expressed under the control of the endogenous *App* locus (Extended Data Fig. 2h). We also tested whether aged myelin mutant animals per se (without the human *APP* transgene) could naturally develop amyloid plaques in their lifetime. No amyloid deposits, however, were detected in 14-month old *Plp*^*−/y*^ mice or in 22-month-old long-lived mice in which *Plp* was conditionally knocked out in the forebrain (*Emx*^*cre*^*Plp*^*fl/fl*^ mice) (Extended Data Fig. 2i,j). These results are in line with studies showing that rodent amyloid is relatively resistant to aggregation^18^ and that mice overexpressing rodent Aβ do not develop amyloidosis in their lifetime^19^.

**Extended Data Fig. 3:**
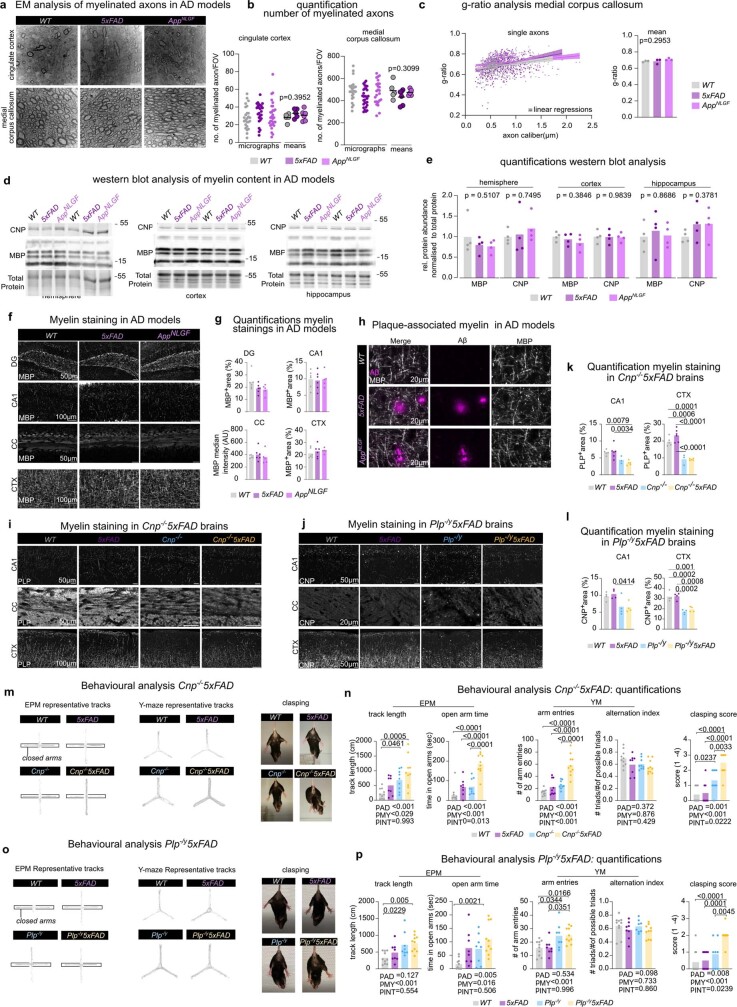
Myelin status in AD mouse models and myelin mutant crossbreedings and their behavioural analysis. (**a**) Representative EM images of regions of the cingulate cortex and medial corpus callosum in *App*^*NLGF*^ and *5xFAD* mice at 6 months of age. (**b**) Quantification of myelinated axon counts per field of view (FOV) of EM images as shown in (a). Dots represent single micrographs analysed. 5 images per animal/biological replicat/n were analysed (n = 5 for *WT* and *App*^*NLGF*^, n = 6 for *5xFAD*). Contoured dots represent the mean for each biological replicate. Statistical analysis: ordinary one-way ANOVA (p-value given in graphs) with Tukey’s multiple comparisons (no significant differences were found). (**c**) Analysis of myelin thickness by g-ratio measurements of single axons in the medial corpus callosum of *WT, 5xFAD* and *App*^*NLGF*^ mice at 6 months of age. Dots represent single axons analysed (*WT* = 477, *5xFAD* = 512, *App*^*NLGF*^ = 533 from 3 animal/biological replicat/n per group); lines represent linear regression, dotted line represent 95% confidence bands. In the bar graph, bars represent means and dots represent biological replicates/mice/n (n = 3). Statistical analysis: ordinary one-way ANOVA (p-value given in graphs) with Tukey’s multiple comparisons. (**d**) Western blot analysis of myelin proteins MBP and CNP in *5xFAD* and *App*^*NLGF*^ mice at 6 months of age in different brain regions (hemisphere, cortex, hippocampus). Molecular weight marker (in kDa) is indicated on the left. (**e**) Quantification of western blot analysis shown in (d). Relative protein abundance was determined by normalisation to total protein staining and wildtype levels. Bars represent means; dots represent biological replicates/mice/n (n = 4 per group). Statistical analysis: ordinary one-way ANOVA per group (antibody, brain region). p-values are given above bar graphs. (**f**) Closeups of myelin (anti-MBP) labelling in 6-months old *WT, 5xFAD* and *App*^*NLGF*^ mice in (DG), cornu ammonis (CA1), corpus callosum (CC), and cortex (CTX). (**g**) Quantification of myelin profiles (MBP^+^ area) in DG, CA1 and CTX and intensity in CC. Bars represent means; dots represent biological replicates/mice/n (n = 6 for *WT*, n = 6 for *5xFAD*, n = 5 for *App*^*NLGF*^). Statistical analysis: ordinary one-way ANOVA with Tukey’s multiple comparison test was performed. No significant (p < 0.05) differences were found. (**h**) Immunofluorescence closeups of myelin (MBP labelled) in plaque proximity (labelled with 6E10) in *WT, 5xFAD* and *App*^*NLGF*^ cortex. The same observations were made in different independent samples/mice per group (n = 6 for *WT*, n = 6 for *5xFAD*, n = 5 for *App*^*NLGF*^). (**i**) Closeups of myelin (anti-PLP) labelling in *Cnp*^*−/−*^*5xFAD* mice, corresponding single mutants (*Cnp*^*−/−*^ and *5xFAD*) and wildtype (*WT*) controls in the CA1, CTX and CC at 6 months of age. (**j**) Closeups of myelin (anti-CNP labelling in *Plp*^*−/y*^*5xFAD* mice, corresponding single mutants (*Plp*^*−/y*^ and *5xFAD*) and wildtype (*WT*) controls in the CA1, CTX and CC region at 6 month of age. (**k**) Quantification of myelin profiles (PLP+ area) in CA1 and CTX in *Cnp*^*−/−*^*5xFAD* brains shown in (i). Bars represent means; dots represent biological replicates/mice/n (n = 5 for *WT*, n = 6 for *5xFAD*, n = 3 for *Cnp*^*−/−*^, n = 5 for *Cnp*^*−/−*^*5xFAD)*. Statistical analysis: ordinary one-way ANOVA with Tukey’s multiple comparison tests. P-values for p < 0.05 are shown in the bar graph. Non-significant p-values are not shown. (**l**) Quantification of myelin profiles (CNP+ area) in CA1 and CTX in *Plp*^*−/y*^*5xFAD* brains shown in (j). Bars represent means; dots represent biological replicates/mice/n (n = 4 per group). Statistical analysis: ordinary one-way ANOVA with Tukey’s multiple comparison tests. P-values for p < 0.05 are shown in the bar graph. Non-significant p-values are not shown. (**m**) Representative tracks and images for the behaviour of *Cnp*^*−/−*^*5xFAD* female mice in the elevated plus maze (EPM), Y maze (YM) and the clasping test paradigms. (**n**) Quantification of the behaviour of *Cnp*^*−/−*^*5xFAD* female mice in the paradigms shown in (m). Bars represent means; dots represent biological replicates/mice/n (n = 10 for *WT*, n = 8 for *5xFAD*, n = 9 for *Cnp*^*−/−*^, n = 10 for *Cnp*^*−/−*^*5xFAD*). (**o**) Representative tracks and images for the behaviour of Plp^*−*/y^5xFAD male mice in the elevated plus maze (EPM), Y maze (YM) and the clasping test paradigms. (**p**) Quantification of the behaviour of *Plp*^*−/−*^*5xFAD* female mice in the paradigms shown in (m). Bars represent means; dots represent biological replicates/mice/n (n = 10 for *WT*, n = 8 for *5xFAD*, n = 8 for *Plp*^*−/y*^, n = 10 for *Plp*^*−/y*^*5xFAD*). Statistical analysis for (**n**) and (**p**): two-way type III ANOVAs probing the main effects for the *5xFAD* genotype (p_AD_) and the respective *myelin-mutant* genotype (p_MY_) as well as their interaction (p_INT_) followed by Tukey’s multiple comparisons posthoc test (p-value is given in the bar graphs). Source data

Secondary myelin alterations have been described in mouse models of AD^20,21^. However, our immunostaining, western blot and electron microscopy (EM) analyses did not detect substantial myelin pathology in 6-month-old 5×FAD mice or *APP*^*NLGF*^ mice (Extended Data Fig. 3). Similarly, the subtle myelin pathology that occurs in *Cnp*^*−/−*^ mice and *Plp*^*−/y*^ mice was not modified by crossbreeding to the 5×FAD genotype (Extended Data Fig. 3i–l). The observed plaque-promoting effects are therefore not simply an altered secondary response of oligodendrocytes or myelin to amyloid deposits.

We also investigated whether myelin dysfunction modifies 5×FAD behavioural deficits by performing behavioural testing in the Y-maze and the elevated plus maze (Extended Data Fig. 3m–p). In both types of mazes, myelin mutant mice presented with hyperactivity that was further exacerbated in *Cnp*^*−/−*^;5×FAD mice. In the elevated plus maze, *Cnp*^*−/−*^5×FAD mice preferred the open arms, which is indicative of an unusual lack of anxiety. Statistical analyses confirmed supra-additive effects (interactions) of 5×FAD and especially the *Cnp*^*−/−*^ genotype. Myelin defects and amyloid pathology seem to synergistically worsen behavioural deficits indicative of disinhibition, which is a neuropsychiatric symptom that is also present in patients with AD^22^.

## Acute demyelination drives Aβ deposition

**Extended Data Fig. 4:**
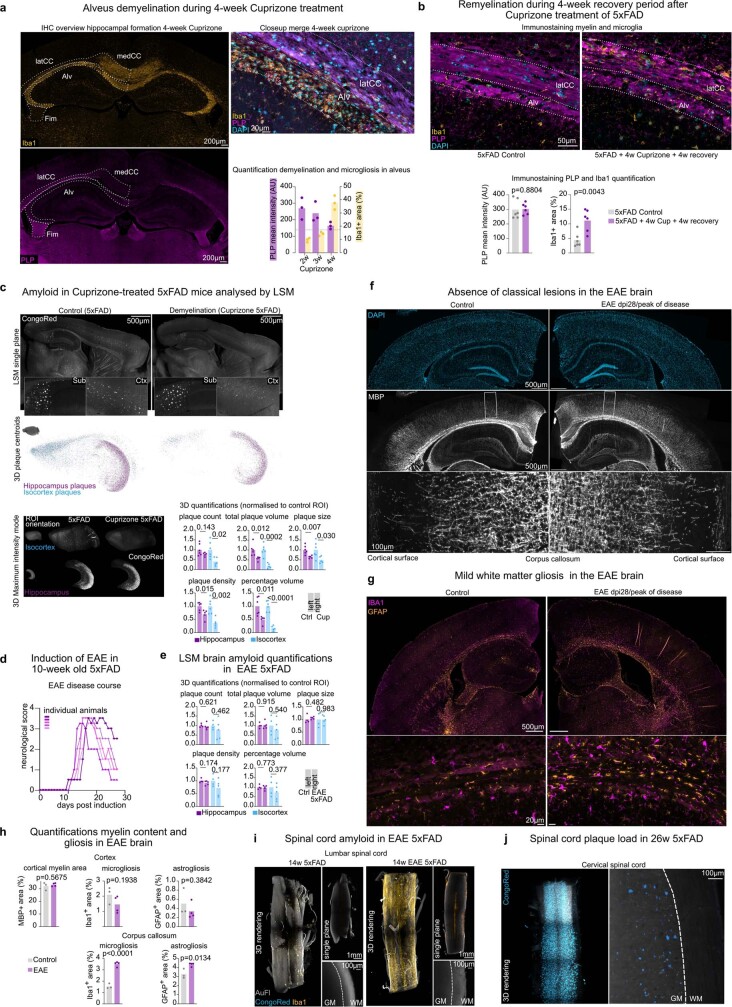
Cuprizone and EAE induced changes in brain myelin status and its influence on brain plaque burden. (**a**) Histological analysis of Cuprizone-induced brain demyelination and microglia/macrophage infiltration after 4-week Cuprizone treatment by Iba1 and PLP immunostaining. Dashed lines border indicated ROIs: latCC = lateral corpus callosum. medCC = medial corpus callosum. Alv = Alveus. Fim = Fimbria. Closeup of immunostaining showing specific demyelination and microglia/macrophage accumulation of the medial corpus callosum and alveus after 4 weeks of Cuprizone treatment. Quantification of immunostaining for PLP (myelin) and microglia/macrophage (Iba1) in the alveus as shown in (a) throughout 2, 3 and 4 weeks of Cuprizone treatment. Bars represent means; dots represent biological replicates/mice/n (n = 3 per group). Dashed line represents mean background fluorescence of PLP staining. (**b**) Histological analysis of remyelination after a 4-week recovery period after Cuprizone withdrawal by Iba1 and PLP immunostaining. Bars represent means; dots represent biological replicates/mice/n (n = 6 per group). FStatistical analysis: two-sided, unpaired Student’s t-test (p-value is given in graph). (**c**) LSM single plane of Congo Red stained brains and closeups of the subiculum and a cortical region. Middle panel shows 3D distribution of isocortical and hippocampal plaques represented as centroids. Lower panel shows 3D maximum intensity projection of cropped regions of interest. Right lower panel shows 3D quantifications of plaque burden parameters in hippocampus and isocortex. Bars represent means; dots represent biological replicates/mice/n (n = 6 for control, n = 5 for Cup). Statistical analysis: two-sided, unpaired Student’s t-test (p-value is given in graph). (**d**) Neurological scoring shows successful EAE-induction in *5xFAD* animals with typical disease onset at around day 10 post induction (dpi 10). Lines represent time courses for single animals/replicates/mice/n (n = 5). Dots represent daily scores. (**e**) 3D quantifications of plaque burden in the brain of EAE *5xFAD* animals 4 weeks dpi (age 14 weeks). Bars represent means; dots represent biological replicates/mice/n (n = 4 for control, n = 5 for EAE *5xFAD*). Statistical analysis: two-sided, unpaired Student’s t-test (p-value is given in graph). (**f**) Immunofluorescence analysis of potential brain lesions in the EAE brain using nuclei staining (DAPI) and anti-MBP staining. No classical lesions (accumulation of nuclei and corresponding loss of myelin) were observed in the brain. Lower panel shows closeup of the area indicated in the middle panel. (**g**) Immunofluorescence analysis of gliosis in the EAE brain using Iba1 and GFAP staining. (**h**) Quantification of myelin content and gliosis (as shown in (f) and (g)) in cortex and corpus callosum of the EAE brain. Bars represent means; dots represent biological replicates/mice/n (n = 3 for control, n = 4 for EAE). Statistical analysis: two-sided, unpaired Student’s t-test (p-value is given in graph). (**i**) Light sheet microscopic analysis of plaque burden in the lumbar spinal cord of EAE *5xFAD* mice. No typical grey matter plaques could be detected in 14 week old *5xFAD* or EAE *5xFAD* mice. The lumbar spinal cord was heavily affected by EAE lesions as visualised by Iba1 staining.The same observations were made in three independent samples/mice per group (n = 4 for control, n = 5 for EAE *5xFAD*). (**j**) As a positive control for successful detection of spinal cord plaques by light sheet microscopy, a cervical spinal cord of a 6-month-old *5xFAD* animal is shown. Source data

**Extended Data Fig. 5:**
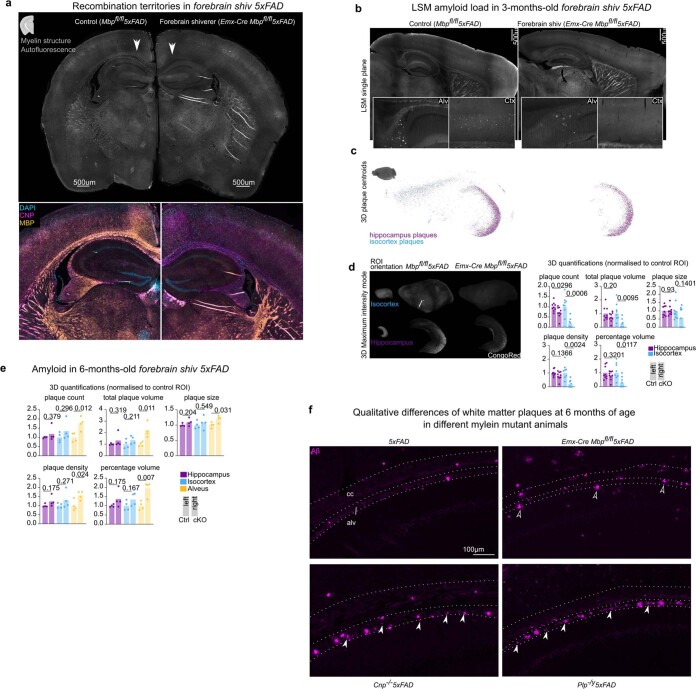
Recombination territories and plaque load in *forebrain shiverer 5xFAD* mice. (**a**) Basic characterisation of myelination in *Emx-Cre Mbp*^*fl/fl*^*5xFAD* mice. Autofluorescence shows a clear lack of myelinated profiles in the corpus callosum (arrows) while the thalamus and striatum show normal myelin profiles. The lower panel shows closeup images of anti-CNP and MBP co-immunolabelling in *Emx-Cre Mbp*^*fl/fl*^*5xFAD*. Lack of myelin compaction (MBP^−^CNP^+^) throughout the cortex, hippocampus and subcortical white matter. Compaction of myelin is unaffected in other brain regions such as the thalamus and striatum (MBP^+^CNP^+^). The same observations were made in 5 independent samples/mice per group. (**b**-**d**) *In toto plaque* load in 3-month-old forebrain *shiverer* 5xFAD (*Emx-Cre Mbp*^*fl/fl*^ *5xFAD*). (**b**) Representative LSM 2D single planes show closeup images of the cortex (Ctx) and alveus (Alv). (**c**) 3D representation of hippocampal and isocortical represented as coloured centroids. (**d**) 3D cropped regions of interest of brains rendered in maximum intensity modus and 3D quantification of plaque load in the three regions of interest normalised to the respective region in control animals. Bars represent means; dots represent biological replicates/mice/n (n = 9 for control, n = 9 for mutant). Statistical analysis: two-sided, unpaired Student’s t-test (p-value is given in graph). (**e**) Quantification of light sheet microscopic analysis of plaque load in 6-month-old *Emx-Cre Mbp*^*fl/fl*^*5xFAD* mice. Bars represent means; dots represent biological replicates/mice/n (n = 4 per group). Statistical analysis: two-sided, unpaired Student’s t-test (p-value is given in graph). (**f**) Immunofluorescence labelling of amyloid plaques in the white matter of 6-month old *5xFAD* control mice and different myelin mutant *5xFAD* crossbreedings. Note the differences of white matter plaques in the amyelination model (*Emx-Cre Mbp*^*fl/fl*^*5xFAD)* versus dysmyelination models *(Cnp*^*−/−*^/*Plp*^*−/y*^ *5xFAD)* in regards to their distribution and appearance: While in *Cnp*^*−/−*^ and *Plp*^*−/y*^ crossbreedings enhanced plaque deposition is restricted to the alveus (two lower dashed lines), in *Emx-Cre Mbp*^*fl/f*^ *5xFAD* mice enhanced plaque deposition can also be seen in the corpus callosum (upper two dashed lines). Plaques in the alveus of *Cnp*^*−/−*^ and *Plp*^*−/y*^ crossbreedings appeared smaller and more numerous (white arrowheads), while alveus plaques in *Emx-Cre Mbp*^*fl/fl*^ appeared bigger in size and more isolated (black contoured arrow heads). cc:corpus callosum, alv: alveus. The same observations were made in three independent samples/mice per group. Source data

To confirm that myelin dysfunction is an upstream driver of plaque pathology, we tested the effect of acute demyelination. Young adult 5×FAD mice were fed a cuprizone-containing diet for 4 weeks followed by a 4-week recovery period, after which the plaque load was determined by LSM. This method leads to substantial demyelination in the medial corpus callosum and the alveus accompanied by microgliosis followed by complete remyelination after 4 weeks (Extended Data Fig. 4a,b). Interpreting the outcome of this experiment was complicated, however, because the copper-chelating properties of cuprizone interfere with plaque core formation, which depends on copper^23^. Indeed, the in toto LSM results showed that cuprizone treatment seemed to ameliorate hippocampal and cortical Aβ pathology (Extended Data Fig. 5c). However, the amyloidosis-driving effect of demyelination predominated the inhibition by copper chelation in substantially demyelinated areas, such as the hippocampal alveus. In this brain region, less-compacted Aβ plaques in cuprizone-treated 5×FAD mice were well stained with anti-Aβ antibodies, which revealed a large increase in small amyloid aggregates (Fig. 2e).

Independent evidence to support our working model was provided by results from 5×FAD mice in which experimental autoimmune encephalomyelitis (EAE) was induced. We immunized young 5×FAD animals with myelin oligodendrocyte glycoprotein (MOG) peptide and analysed their brains and spinal cords 4 weeks later (Fig. 2f and Extended Data Fig. 4d). Results from a previous study^24^ of aged J20 and Tg2576 mouse models of AD showed that EAE reportedly reduced brain plaque load. However, we did not find a difference in amyloid deposition in young 5×FAD mice with EAE (Extended Data Fig. 4e) due to the lack of demyelination pathology in the brain (Extended Data Fig. 4f–h). By contrast, in the spinal cord, where demyelinating EAE pathology substantially develops (Extended Data Fig. 4i), we identified small, atypical amyloid aggregates in the peri-lesion environment, which were absent from control 5×FAD mice (Fig. 2f). We verified the presence of aggregated amyloid by staining spinal cord sections with the β-sheet dye methoxy-04 (Me-04) (Fig. 2f). Of note, in spinal cord from WT animals with EAE, no such Me-04^+^ material was found, which rules out the possibility of nonspecific detection of lipid deposits in demyelinated lesions (Fig. 2f). Spinal cord grey matter plaque deposition, as seen later in 5×FAD mice with disease progression, could not be detected in 14-week-old 5×FAD mice or in EAE 5×FAD mice (Extended Data Fig. 4i,j). Taken together, myelin defects—both chronic and acute—drive amyloid deposition in mouse models of AD, which identifies dysfunctional myelin as an upstream risk factor for amyloid deposition.

## Lack of myelin ameliorates Aβ deposition

Compared to other apes, humans show a disproportional enlargement of prefrontal white matter^25^. This raises the question of whether the extent of cortical myelination per se could have a causal role in human AD. We therefore wondered what the impact of the near complete absence of cortical myelin would have on the course of amyloidosis in 5×FAD mice. To this end, we generated a line of forebrain-specific shiverer mice in which cortical axons are largely unmyelinated (*Emx*^*cre*^*Mbp*^*fl/fl*^; forebrain shiverer mice) and crossbred them to 5×FAD mice (Extended Data Fig. 5a). At 3 months of age, forebrain shiverer;5×FAD mice were strongly protected against amyloid deposition in both the hippocampus and the cortex (Extended Data Fig. 5b–d). However, at 6 months of age, this effect was largely lost, which indicated that there is a delay in plaque formation in the absence of myelin (Extended Data Fig. 5e). At 6 months of age, plaque burden in the alveus was increased in forebrain shiverer;5×FAD mice, quantitatively similar to *Cnp*^*−/−*^ and *Plp*^*−/y*^ crossbred mice. Qualitatively, however, alveus plaques in forebrain shiverer;5×FAD mice differed in their morphology and distribution (Extended Data Fig. 5f), which pointed towards a different mechanism of increased plaque burden in these models. We conclude that proper myelin ensheathment and healthy myelin have an initially inhibitory effect on plaque formation.

## Myelin dysfunction affects APP metabolism

**Fig. 3:**
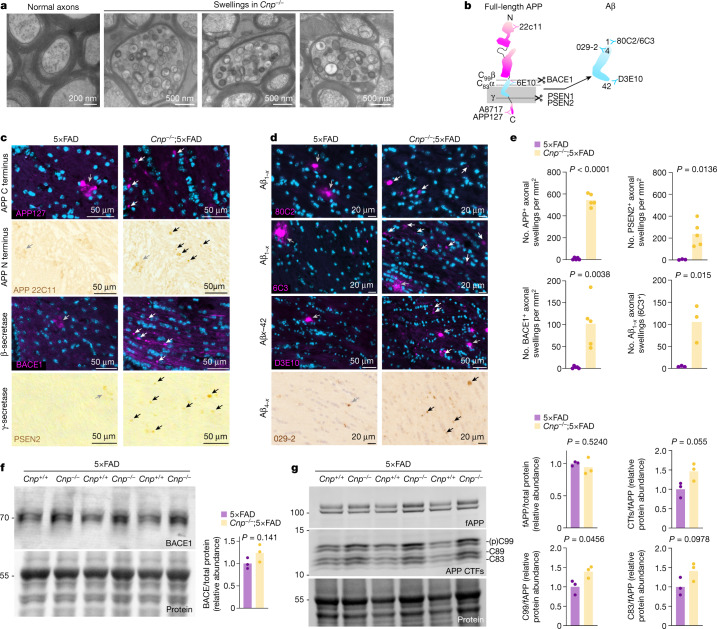
Myelin dysfunction alters APP processing. **a**, High-pressure freezing EM of optic nerves from 6-month-old WT and *Cnp*^*−/−*^ mice. Nerves from *Cnp*^*−/−*^ mice show (plaque-independent) axonal swellings with accumulation of endosomal and lysosomal structures and multivesicular bodies. The same observations were made in three independent samples per mice per group. **b**, Schematic representation of APP cleavage and binding sites of anti-APP and anti-Aβ antibodies used. **c**, Fluorescent and chromogenic immunostaining images of APP and APP cleavage enzymes BACE1 (β-secretase) and PSEN2 (as part of the γ-secretase complex) in white matter (fimbria) from *Cnp*^*−/−*^;5×FAD mice. Grey arrowheads mark plaque-associated axonal swellings typically forming a corona. Black and white arrowheads indicate plaque-independent axonal swellings as observed in *Cnp*^*−/−*^ mice. **d**, Fluorescent and chromogenic immunostainings of Aβ peptides in white matter (fimbria) of *Cnp*^*−/−*^;5×FAD mice and 5×FAD mice. Grey arrowheads indicate proper amyloid plaques, typically stained intensely. White arrows indicate swellings stained positive by the respective Aβ-antibody, but typically stained less intensely and more round in structure. **e**, Quantification of axonal swellings positive for APP, BACE1, PSEN2 and Aβ (6C3) in white matter (fimbria) of 5×FAD control mice and *Cnp*^*−/−*^;5xFAD mice. For BACE1 and APP quantification, *n* = 5 for 5×FAD and *Cnp*^*−/−*^;5×FAD. For PSEN2 quantification, *n* = 3 for 5×FAD and *n* = 5 for *Cnp*^*−/−*^;5×FAD. For 6C3, *n* = 3 for 5×FAD and *Cnp*^*−/−*^;5×FAD. **f**, Fluorescent immunoblot analysis of BACE1 levels in microdissected cortex of *Cnp*^*−/−*^;5×FAD mice and 5×FAD mice. **g**, Fluorescent immunoblot analysis of APP fragmentation in the membrane-bound fraction of microdissected cortical tissue of *Cnp*^*−/−*^;5×FAD mice and 5×FAD control mice. (p)C99, phospho-C99. For **f** and **g**, the molecular weight marker (in kDa) is indicated on the left. For quantifications in **f** and **g**, full-length APP (fAPP) and BACE1 levels were normalized to total protein stain. CTF levels were normalized to fAPP. *n* = 3 per group. For **e**–**g**, statistical analysis: two-sided, unpaired Student’s *t*-test (*P* values are indicated in the graphs). Bars represent means, dots represent biological replicates/mice/lanes/*n*. Source data are given in Supplementary Fig. 1. Source data

**Extended Data Fig. 6:**
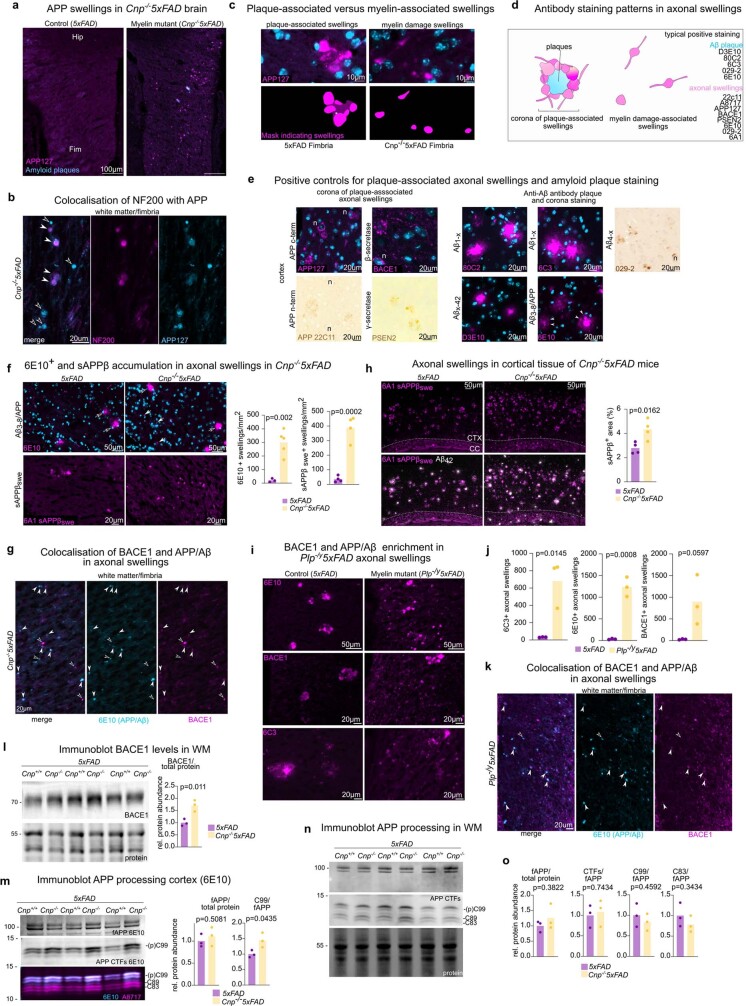
Myelin dysfunction alters APP processing in *Cnp*^*−/−*^*5xFAD and Plp*^*−/y*^*5xFAD* mice. Continuation from Fig. 3. (**a**) Overview of APP-stained swellings in the fimbria of *5xFAD* control and *Cnp*^*−/−*^*5xFAD* mice. Hip: Hippocampus. Fim: Fimbria. The same observations were made in 5 independent samples/mice per group. (**b**) Colocalisation of APP and NF200 (axonal cytoskeletal marker) in swellings confirming their axonal origin in *Cnp*^*−/−*^*5xFAD* mice. White arrowheads indicate swellings double-positive for APP and NF200. Black-contured arrows indicate swellings that are solely positive for APP. This is likely a result of cytoskeletal breakdown in axonal swellings. The same observations were made in 3 independent samples/mice per group. (**c**) Distinction of plaque-associated axonal swellings induced by the *5xFAD* genotype and myelin-damage-associated swellings induced by the *Cnp*^*−/−*^ genotype. Upper panels are closeups of images shown in Fig. 3c. Lower panels show a mask indicating axonal swellings. Note that plaque-associated swellings form a circular arrangement of multiple swellings (corona) around a plaque centre (asteriks). In contrast, myelin damage-associated axonal swellings occur isolated/scattered. (**d**) Schematic representation of plaque-associated axonal swellings and myelin damage-associated swellings, and typical stainings observed with antibodies shown in Fig. 3b. D3E10, 80C2 and 6C3 do not show cross-reactivity to full length APP and typically only very weakly stain APP-positive plaque-associated swellings, but robustly stain swellings in *Cnp*^*−/−*^*5xFAD* mice. (**e**) Left panels shows positive staining controls for APP processing machinery antibodies used in Fig. 3 in the *5xFAD* grey matter. For APP, BACE1 and PSEN2 typical plaque-corona staining (contoured arrowheads) can be observed. For APP and BACE1 additional neuronal cell body staining (n) can be observed. Right panel shows positive staining controls for amyloid antibodies used in Fig. 3 in the *5xFAD* grey matter. Contoured arrowheads indicate proper amyloid plaques, typically stained very intensely. 029-2 and 6E10 antibodies show cross-reactivity to full-length APP and also stain plaque-associated axonal swellings (white arrowheads) and neurons (n). (**f**) 6E10 and sAPPβ_swe_ (soluble cleavage product after β-cleavage of APP) labelling of axonal swellings in *Cnp*^*−/−*^*5xFAD* fimbria. Figure 4d continued. Quantifications are shown on the right. Bars represent means; dots represent biological replicates/mice (for sAPPβ_swe,_ n = 4 for each group; for 6E10 labelling, n = 3 for *5xFAD* controls and n = 5 for *Cnp*^*−/−*^*5xFAD*). Statistical analysis: two-sided, unpaired Student’s t-test (p-value is given in graphs). (**g**) Colocalisation of BACE1 and APP/Aβ in axonal swellings in *Cnp*^*−/−*^*5xFAD* white matter/fimbria. APP/Aβ was visualised using the 6E10 antibody. White arrowheads indicate swellings that are double-positive (to various degrees) for BACE1 and 6E10. Contoured black arrowheads indicate swellings single-positive for either 6E10 or BACE1. The same observations were made in 3 independent samples/mice per group. (**h**) sAPPβ_swe_ staining in cortical tissue of *Cnp*^*−/−*^*5xFAD* mice and *5xFAD* controls. Staining reveals abundant plaque-associated swellings arranged in a swelling corona in 5xFAD mice. In *Cnp*^*−/−*^*5xFAD* both plaque-associated swellings and numerous plaque-independent swellings (i.e. myelin-damage-associated swellings) can be found. Quantification of sAPPβ_swe_ covered area is shown on the right. Bars represent means; dots represent biological replicates/mice/n (n = 4 per group). Statistical analysis: two-sided, unpaired Student’s t-test (p-value is given in graph). (**i**) Immunostaining analysis of APP/Amyloid-β and BACE1 in axonal swellings in *Plp*^*−/y*^*5xFAD* white matter (fimbria). (**j**) Quantifications of immunostainings shown in (i). Bars represent means; dots represent biological replicates/mice/n (n = 3 per group). Statistical analysis: two-sided, unpaired Student’s t-test (p-value is given in graph). (**k**) Colocalisation of BACE1 and APP/Aβ in axonal swellings in *Plp*^*−/y*^*5xFAD* mice. White arrows indicate a number of swellings in which colocalisation occurs. Contoured black arrows indicate swellings without colocalisation. The same observations were made in 3 independent samples/mice per group. (**l**) Fluorescent immunoblot analysis of BACE1 levels in the micro-dissected white matter of *Cnp*^*−/−*^*5xFAD* and *5xFAD* mice. Molecular weight marker (in kDa) is indicated on the left. Bars represent means; dots represent biological replicates/lanes/mice/n (n = 3 per group). Statistical analysis: two-sided, unpaired Student’s t-test (p-value is given in graph). Source data are given in Supplementary Fig. 1. (**m**) Immunoblot analysis of APP processing using the 6E10 antibody. Molecular weight marker (in kDa) is indicated on the left. Anti-c-terminal APP labelling was applied to identify different APP CTF fragments: (p)C99 6E10 and A8717 double-positive; C89 and C83 6E10 negative and A8717 positive. Bars represent means; dots represent biological replicates/lanes/mice/n (n = 3 per group). Statistical analysis: two-sided, unpaired Student’s t-test (p-value is given in graph). Source data are given in Supplementary Fig. 3. (**n**) Fluorescent immunoblot analysis of APP fragmentation in the membrane-bound fraction of micro-dissected white matter (corpus callosum + alveus) of *Cnp*^*−/−*^ *5xFAD* and *5xFAD* control mice. Molecular weight marker (in kDa) is indicated on the left. (**o**) Quantification of western blot analysis shown in (n). Bars represent means; dots represent biological replicates/lanes/mice/n (n = 3 per group). Statistical analysis: two-sided, unpaired Student’s t-test (p-value is given in graph). Source data are given in Supplementary Fig. 1. Source data

We next investigated how myelin defects mechanistically drive amyloidosis. In theory, the defects could either promote APP processing and Aβ generation or interfere with Aβ removal (or a combination of both). We first investigated APP metabolism in *Cnp*^*−/−*^;5×FAD mice. Axonal swellings that stain positive for APP are prominent features of ischaemia, brain injury and myelin disorders, and we speculated that these swellings contribute to the generation of Aβ in myelin mutant animals. Stalling axonal transport can enhance amyloid production, probably by increasing BACE1 and APP interactions in axonally transported vesicles^26–28^, and axons seem to be important sites of amyloid secretion^29–31^. In *Cnp*^*−/−*^ mice and *Cnp*^*−/−*^;5×FAD crossbred mice, numerous myelin damage-associated swellings of axonal origin were observed (Extended Data Fig. 6a–d). In 5×FAD control mice, almost no such swellings were found and axonal injury was limited to the immediate vicinity of the plaque and arranged in a circular pattern (termed corona) (Extended Data Fig. 6a–d). High-pressure freezing EM analyses showed that myelin-damage-associated swellings were enriched in vesicular structures that were probably endosomal or lysosomal in origin (Fig. 3a), which are thought to be primary production sites of Aβ^32,33^. Using antibodies against APP processing enzymes and multiple APP- and Aβ-specific antibodies (Fig. 3b), we found that axonal swellings in the brains of *Cnp*^*−/−*^;5×FAD mice accumulated β-secretase and γ-secretase, co-stain for APP and BACE1 and consequently stain positive for β- and γ-cleaved APP fragments and Aβ (Fig. 3c–e and Extended Data Fig. 6e–g). Immunostaining also showed increased levels of soluble APPβ in cortical tissue in *Cnp*^*−/−*^;5×FAD mice. In this brain region, similar to white matter, numerous myelin-damage-associated swellings were observed in addition to plaque-associated swellings (Extended Data Fig. 6h). Similar observations were made in *Plp*^*−/y*^;5×FAD mice, which were used as an independent model of myelin dysfunction (Extended Data Fig. 6i–k).

We further corroborated these findings by western blot analyses of white matter and the cortex, which showed increased levels of BACE1 (which was significant in white matter) (Fig. 3f and Extended Data Fig. 6l) in 6-month-old *Cnp*^*−/−*^;5×FAD mice. When we investigated APP processing by immunoblotting, cortical but not white matter APP metabolism was shifted towards an increased abundance of APP carboxy-terminal fragments (CTFs), but without changes to full-length APP abundance or the α/β CTF ratio (Fig. 3g and Extended Data Fig. 6m–o). It is tempting to speculate that axonally generated CTFs in the white matter could be retrogradely transported and accumulate over time in neuronal cell bodies of the cortex, as western blotting showed that these areas are enriched in CTFs. Together, these findings suggest that myelin defects affect APP metabolism and Aβ generation.

## Myelin defects alter microglia responses

**Fig. 4:**
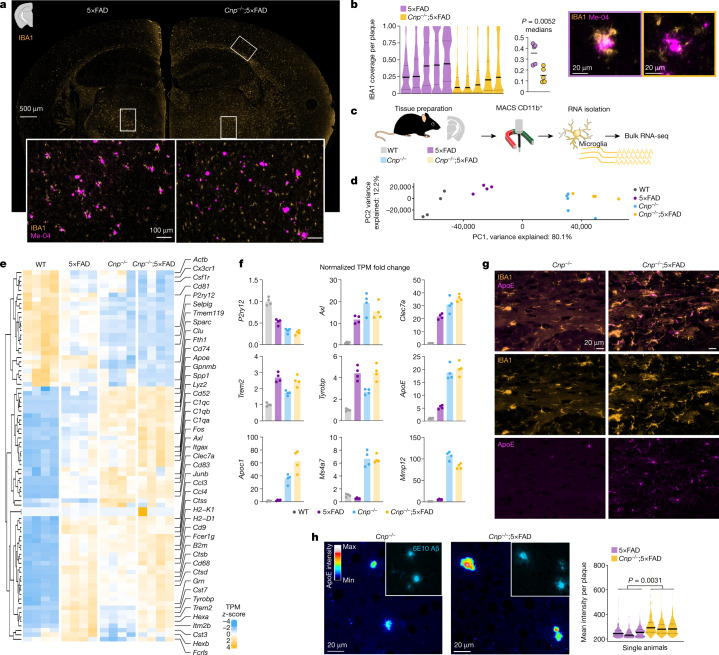
Loss of microglial corralling around amyloid plaques in myelin mutant mice. **a**, 2D immunofluorescence analysis of microglial reaction to amyloid plaques in *Cnp*^*−/−*^;5×FAD and 5×FAD mice by IBA1 and amyloid co-staining (Me-04). **b**, Left, automated quantification of IBA1 plaque coverage in the cortex. Each violin plot represents a single animal/biological replicate/*n*. *n* = 5 per group. Black lines represent medians. In total, 2,017 individual cortical plaques were analysed in 5×FAD brain slices and 2,190 in *Cnp*^*−/−*^;5×FAD brain slices. Middle, graph shows distribution of medians. Lines indicate means. Statistical analysis: two-sided, unpaired Student’s *t*-test (*P* values are indicated on the graphs) on biological replicate data. Right, two representative plaques for each genotype. **c**, Experimental setup for microglia bulk RNA-seq. Microglia were isolated from hemispheres of 6-month-old WT, *Cnp*^*−/−*^, 5×FAD and *Cnp*^*−/−*^;5×FAD animals and subjected to RNA-seq (biological replicates, *n* = 4 for each genotype). **d**, PCA was used for evaluating relative distances between normalized RNA transcripts per kilobase million (TPM) profiles. Dots represent biological replicates. Principal component 1 (PC1) explained 80.1% of data variability and strongly reflected *Cnp*^*−/−*^-dominated microglia transcriptome changes. **e**, Heatmap of top genes contributing to PC1 variability. **f**, Normalized expression level for selected genes (homeostatic marker *P2ry12*, DAM signature *Trem2*, *Tyrobp*, *Axl* and *Clec7a*, and differential regulated genes in *Cnp*^*−/−*^;5×FAD mice (*Apoc1*, *ApoE, Ms4a7* and *Mmp12*)). Bars represent means, dots represent mice/biological replicates/*n* (*n* = 4 per group). **g**, ApoE enrichment in microglia in white matter from *Cnp*^*−/−*^;5×FAD mice. Immunofluorescence staining of ApoE and IBA1 to mark microglia. The same observations were made in three independent samples per mice per group. **h**, Left, microscopy analysis of plaque-associated ApoE in cortical plaques in 5×FAD mice and *Cnp*^*−/−*^;5xFAD mice pseudo-coloured according to a rainbow lookup table. Right, violin plots of mean ApoE fluorescence intensity per plaque. Each violin plot represents a single animal. Black lines indicate medians. Statistical analysis: unpaired, two-tailed Student’s *t*-test on biological replicate data. *n* = 3 per genotype, 703 plaques for 5×FAD, 846 plaques for *Cnp*^*−/−*^;5×FAD in total. Source data

**Extended Data Fig. 7:**
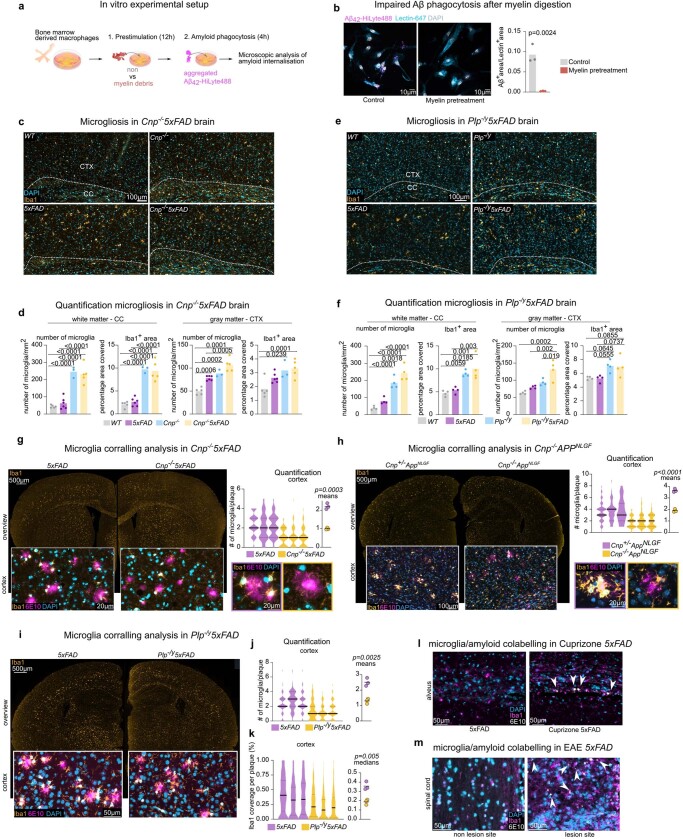
*In vitro* and *in vivo* analysis of microglia challenged by myelin and amyloid. (**a**) Scheme illustrating the experimental setup to study amyloid phagocytosis after myelin digestion. Bone marrow-derived macrophages (BMDMs) were isolated and treated with myelin debris for 12h (1) prior to incubation with HiLyte488-labelled Aβ_42_ for 4h (2). The internalisation of Aβ_42_ was assessed by microscopy. (**b**) Representative immunofluorescence images of BMDMs treated with HiLyte488-labelled Aβ_42_ with and without (control) prior myelin treatment. Quantification of amyloid internalisation normalised to Lectin^+^ area is given on the right. Bars represent means; dots represent replicates/coverslips/n. (n = 3 per group, with 2-3 quantified images per coverslips). Statistical analysis: two-sided, unpaired Student’s t-test (p-value is given in graph). (**c**) Representative images of microglia immunostainings (Iba1 and DAPI) in 6-months old *Cnp*^*−/−*^*5xFAD* mice and respective controls (*WT, Cnp*^*−/−*^ and *5xFAD*). Dotted line highlights the border between cortical (CTX) and callosal tissue (CC). (**d**) Quantification of microgliosis (number of microglia, Iba1^+^ area) in 6-months old *Cnp*^*−/−*^*5xFAD* mice and respective controls (*WT, Cnp*^*−/−*^ and *5xFAD*) in the corpus callosum (CC) and cortex (CTX). Bars represent means; dots represent biological replicates/mice (n = 5 for *WT*, n = 6 for *5xFAD*, n = 3 for *Cnp*^*−/−*^, n = 5 for *Cnp*^*−/−*^*5xFAD*) Statistical analysis: ordinary one-way ANOVA with Tukey’s posthoc test for multiple comparisons (p-values for the posthoc test for all p < 0.05 are given in the graphs). (**e**) Representative images of microglia immunostainings (Iba1 and DAPI) in 6-months old *Plp*^*−/y*^*5xFAD* mice and respective controls (*WT*, *Plp*^*−/y*^ and *5xFAD*). Dotted line highlights the border between cortical (CTX) and callosal tissue (CC). (**f**) Quantification of microgliosis (number of microglia, Iba1^+^ area) in 6-months old *Plp*^*−/y*^*5xFAD* mice and respective controls (*WT*, *Plp*^*−/y*^ and *5xFAD*) in the corpus callosum (CC) and cortex (CTX). Bars represent means; dots represent biological replicates/mice (n = 4 for each experimental group). Statistical analysis: ordinary one-way ANOVA with Tukey’s posthoc test for multiple comparisons (p-values for the posthoc test for all p < 0.1 are given in the graphs). (**g**) Microscopic analysis of microglial corralling by amyloid (6E10), microglia (Iba1) and nucleus (DAPI) co-labelling in 6-month-old *Cnp*^*−/−*^*5xFAD* and *5xFAD* control mice. Upper panel: overview images of the Iba1 staining. Lower panel: closeup of a cortical region. Quantification of microglia corralling (number of microglia per plaque) in the cortex is given on the right. Violin plots show the distributions for single biological replicates/mice/n (n = 3 per genotype, 50 plaques per replicate). Black lines in violin plots indicate medians. Bar graph on the right shows the comparison of means (n = 3 per genotype, 50 plaques per replicate). Statistical analysis: Two-sided, unpaired Student’s t-test (p-value is given above graph). Right, lower panel: closeup of a representative microglia-plaque interaction in each genotype. (**h**) Microscopic analysis of microglial corralling by amyloid (6E10), microglia (Iba1) and nucleus (DAPI) co-labelling in 6-month-old *Cnp*^*−/−*^*App*^*NLGF*^ and *Cnp*^*+/−*^*App*^*NLGF*^ control mice. Upper panel: overview images of the Iba1 staining. Lower panel: closeup of a cortical region. Quantification of microglia corralling (number of microglia per plaque) in the cortex of *Cnp*^*−/−*^*App*^*NLGF*^ and *Cnp*^*+/−*^*App*^*NLGF*^. Violin plots show the distributions for single biological replicates/mice/n (n = 3 per genotype, 50 plaques per replicate). Black lines in violin plots indicate medians. Bar graph on the right shows the comparison of means (n = 3 per genotype, 50 plaques per replicate). Line indicates means. Statistical analysis: Two-sided, unpaired Student’s t-test (p-value is given above graph). Right, lower panel: closeup of a representative microglia-plaque interaction per genotype. (**i**) Microscopic analysis of microglial corralling by amyloid (6E10), microglia (Iba1) and nucleus (DAPI) co-labelling in 6-month-old *Plp*^*−/y*^*5xFAD* and *5xFAD* control mice. Upper panel: overview images of the Iba1 staining. Lower panel: closeup of a cortical region. (**j**) Quantification of microglia corralling (number of microglia per plaque) in the cortex of 6-month old *Plp*^*−/y*^*5xFAD* and *5xFAD*. Violin plots show the distributions for single biological replicates/mice/n (n = 3 per genotype, 50 plaques per replicate). Black line in violin plots indicate medians. Bar graph on the right shows the comparison of means (n = 3 per genotype, 50 plaques per replicate). Line indicates mean. Statistical analysis: Two-sided, unpaired Student’s t-test (p-value is given above graph). (**k**) Quantifications of microglia corralling (microglia coverage per plaque) in the cortex of 6-month-old *Plp*^*−/y*^*5xFAD* and *5xFAD*. Violin plots show the distributions for single biological replicates/mice/n (n = 3 per genotype; 2010 plaques for *5xFAD* and 2529 plaques for *Plp*^*−/y*^*5xFAD* genotype in total). Black line in violin plots indicate medians. Bar graph on the right shows the comparison of medians (n = 3 per genotype). Line indicates mean. Statistical analysis: Two-sided, unpaired Student’s t-test (p-value is given above graph). (**l**) Microscopic analysis of microglial corralling in Cuprizone-treated *5xFAD* animals by amyloid (6E10), microglia (Iba1) and nucleus (DAPI) co-labelling. Note residual white matter gliosis in Cuprizone *5xFAD* mice and small amyloid deposits highlighted by white arrowheads. The same observations were made in different independent samples/mice per group (n = 4 for *5xFAD* and n = 5 for Cuprizone *5xFAD)*. (**m**) Microscopic analysis of microglial corralling in EAE *5xFAD* animals by amyloid (6E10), microglia (Iba1) and nucleus (DAPI) co-labelling. A comparison of a non-lesion site versus a lesion site is shown. Note the massive microglia/macrophage infiltration at the lesion site that made corralling analysis difficult. The same observations were made in different independent samples/mice per group (n = 5 for *5xFAD* and n = 5 for Cuprizone *5xFAD)*. Source data

Glial cells play important parts in the clearance of myelin debris and amyloid peptides, and microglia form barriers around amyloid plaques^34^. Thus, we investigated in vitro and in vivo how these cells react to amyloid when additionally challenged with defective myelin. Prior exposure of bone-marrow-derived macrophages—a commonly used in vitro model to study AD-related phagocyte function—to myelin debris strongly inhibited amyloid phagocytosis (Extended Data Fig. 7a,b). In vivo, cortical microglia failed to cluster around amyloid plaques in *Cnp*^*−/−*^;5×FAD mice and *Plp*^*−/y*^5×FAD mice despite substantial increases in microglia numbers in both white and grey matter in these models (Fig. 4a–d and Extended Data Fig. 7c–k). Notably, we observed the same effect in *Cnp*^*−/−*^;*APP*^*NLGF*^ crossbred mice (Extended Data Fig. 7h). An equivalent analysis of this phenotype was not possible in the primary demyelination models owing to substantial microglia and macrophage infiltration and because the primary effects in these models were not observed in cortical tissue (Extended Data Fig. 7l,m).

**Extended Data Fig. 8:**
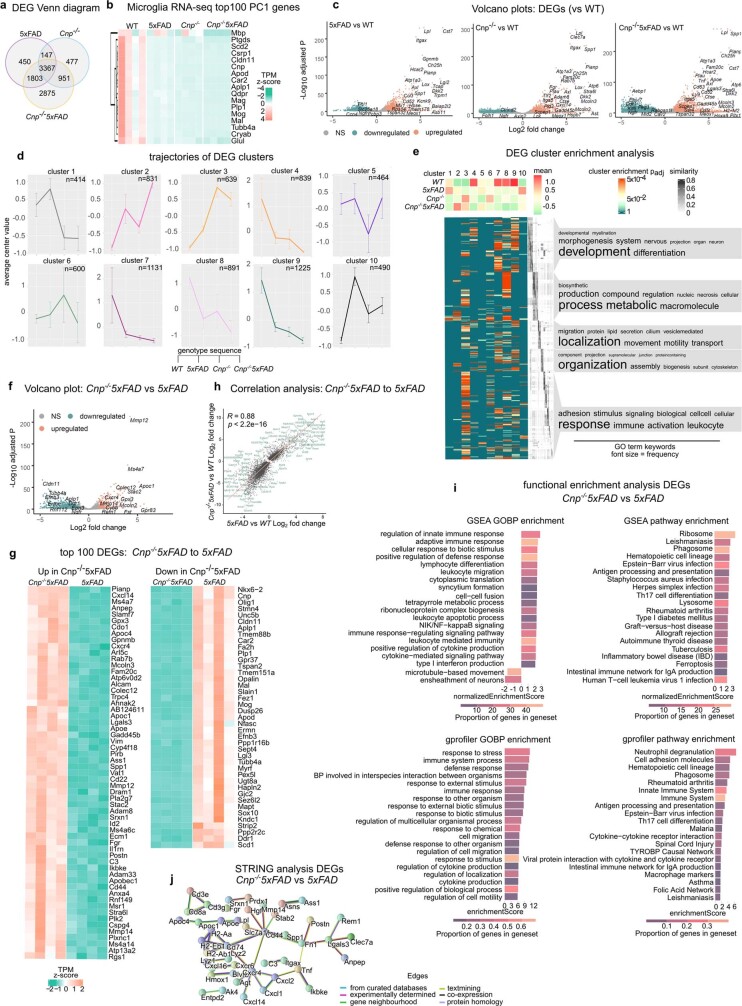
Analysis of microglia bulk RNA sequencing data from *Cnp*^*−/−*^*5xFAD*, single mutants and *WT* controls. Extension of Fig. 4. (**a**) Venn diagram presentation of shared/unique differentially expressed genes (DEGs) between experimental groups (compared to *WT*). (**b**) Myelin-related transcript cluster in the top100 gene list contributing to principal component 1 (PC1). Note that the detection of myelin-related transcripts most likely results from myelin contamination in the CD11b-MACS purified fraction. Decreased contamination (i.e. higher purity) in *5xFAD*, *Cnp*^*−/−*^ and *Cnp*^*−/−*^*5xFAD* is presumably linked to the increased number of microglia cells and advanced tissue disintegration in inflammatory conditions. Each heatmap tile represents a single animal/biological replicate/n (n = 4 per genotype). (**c**) Volcano plot representation of differentially expressed genes in single mutants (*5xFAD*, *Cnp*^*−/−*^) and double mutant *Cnp*^*−/−*^*5xFAD* in comparison to *WT* animals (significant cutoff adjP < 0.01, log2FC > 1.0), (**d**) Line plots for the 10 DEG groups identified by k-means clustering (based on scaled TPM values) with unique trajectories throughout genotypes/experimental groups. Lines represent connections between individual genotype data. Error bars represent SD. (**e**) Heatmap of normalised mean expression pattern in the 10 DEG clusters across different genotypes. Each cluster of genes underwent GO enrichment analysis using gprofiler2, and significantly enriched terms were analysed for their similarities. The left-side coloured heatmap indicates GO term significant levels across each cluster. The adjacent grayscale heatmap shows cluster similarities based on their enriched GO terms. Most representative keywords shared by enriched GO terms are given in the text boxes on the right side. (**f**) Volcano plot representation of differentially expressed genes between *Cnp*^*−/−*^*5xFAD* and *5xFAD* microglia (significant cutoff adjP < 0.01, log2FC > 1.0). (**g**) Top100 differentially regulated genes (based on significant levels) between *Cnp*^*−/−*^*5xFAD* and *5xFAD* microglia. Left panel shows genes upregulated in *Cnp*^*−/−*^*5xFAD*. Right panel shows genes downregulated in *Cnp*^*−/−*^*5xFAD*. (**h**) Correlation analysis of DEGs in *Cnp*^*−/−*^*5xFAD* and *5xFAD* in comparison to *WT*, respectively, reveals highly correlated gene regulations. Pearson correlation coefficient (R) and corresponding p-value is given in the graph. (**i**) Functional enrichment analysis of DEGs from *Cnp*^*−/−*^*5xFAD* vs *5xFAD* using gprofiler and gene set enrichment analysis (GSEA). For each analysis, gene ontology biological process and pathway enrichment is given, and the top 20 significant terms are presented as barplots. The colour indicates the proportion of genes involved in each enriched term. (**j**) STRING interaction analysis of DEGs (significant cutoff adjP < 0.01) between *Cnp*^*−/−*^*5xFAD* and *5xFAD* (confidence 0.9, n = 219 nodes with connections are displayed). The colours of the edges indicate interaction form. Source data

**Extended Data Fig. 9:**
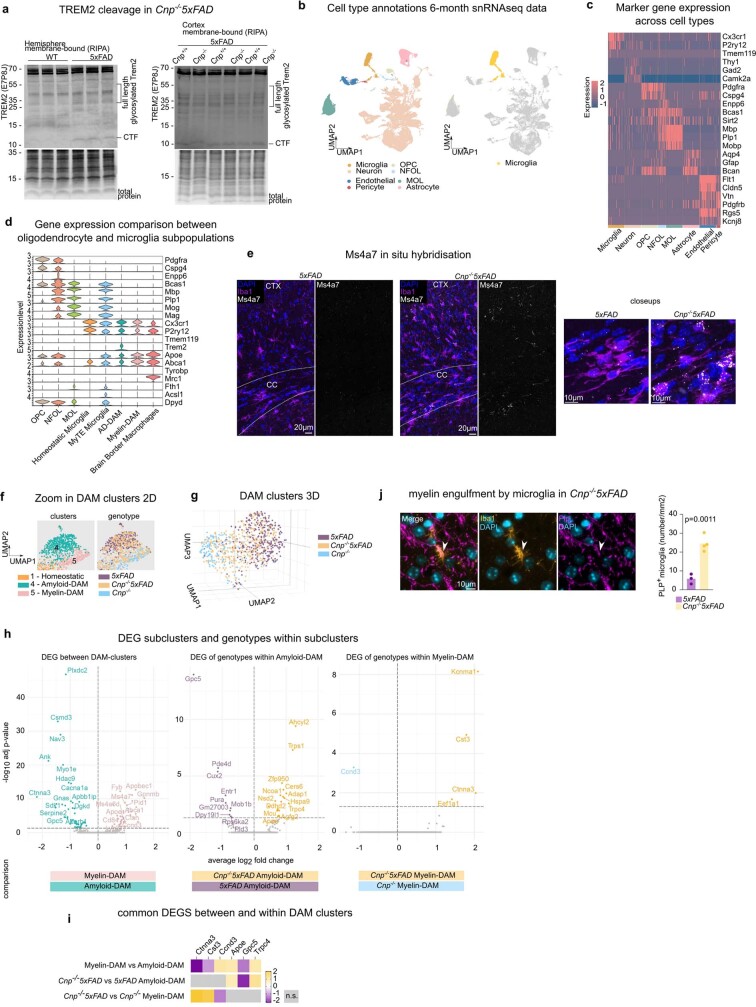
snRNA-seq analysis of *Cnp*^**−/−**^*5xFAD* and respective single mutant controls. Continuation of Fig. 5. (**a**) Left panel shows Western blot analysis of Trem2 levels in *WT vs 5xFAD* mice demonstrating clear induction of full length Trem2 (30–60 kDa, glycosylated forms) and enhanced Trem2 cleavage (~12 kDa) in *5xFAD* mice. Lanes present single animals/biological replicates/n (n = 3 per group). The right panel shows Western blot analysis of Trem2 levels in *Cnp*^*−/−*^ *5xFAD* and *5xFAD* mice. No differences in Trem2 cleavage could be detected. Total protein fastgreen staining is shown as loading control. Lanes present single animals/biological replicates/n (n = 3 per group). Source data are given in Supplementary Fig. 3. (**b**) Cell type distribution and annotations from 6-month-old mouse brain hemisphere snRNA-seq data visualised in the UMAP space. In total 4 genotypes (*WT, Cnp*^*−/−*^, *5xFAD*, and *Cnp*^*−/−*^*5xFAD*) were included and 61,949 cells passed quality controls for downstream analysis. Right panel shows microglial cells highlighted in yellow in UMAP space. (**c**) Heatmap visualisation of scaled marker gene expressions at single-cell level. For each cell population, a random n = 1000 cells were selected to be displayed. For NFOL and pericyte, all cells are shown (total cell numbers from both populations were under 1000). (**d**) Violin plots showing gene expressions across oligodendroglia and microglia subpopulations (microglia detailed subpopulations annotated in Fig. 5b). (**e**) *Ms4a7* fluorescent single molecule in situ hybridisation (RNAscope) in combination with Iba1 immunostaining on *5xFAD* and *Cnp*^*−/−*^*5xFAD* brain tissue. The same observations were made in different independent samples/mice per group (n = 3 per group). (**f**) 2D zoom in on UMAP representation of amyloid and myelin DAM colour coded to cluster annotation (left panel) or genotype (right panel). (**g**) 3D representation of DAM clusters colour-coded according to genotype. (**h**) Volcano plot representation of DEGs between DAM subclusters and between genotypes within Amyloid- and Myelin-DAM clusters. (**i**) Heatmap representation of genes commonly regulated in-between DAM clusters and between genotypes in specific DAM clusters. (**j**) Immunofluorescence analysis of myelin phagocytosis (PLP^+^) by microglia (Iba1^+^) in the cortex of *Cnp*^*−/−*^*5xFAD* mice. Quantification on the right shows the number of myelin-positive(PLP^+^) microglia (Iba1^+^). Bars represent means, dots represent single mice/biological replicates/n (n = 3 for *5xFAD*, n = 4 for *Cnp*^*−/−*^*5xFAD*). Statistical analysis: Two-sided, unpaired Student’s t-test (p-value is given in the graph). Source data

Similar defects in microglial plaque-corralling have been previously described in *Trem2* loss-of-function scenarios^34–36^. We therefore investigated whether myelin dysfunction interferes with the upregulation of *Trem2* and the induction of the disease-associated microglia (DAM) signature that is associated with a plaque-corralling phenotype^37^. To this end, we performed magnetic activated cell sorting (MACS) of microglia and bulk RNA sequencing (RNA-seq) (Fig. 4c). Principal component analysis (PCA) (Fig. 4d and Extended Data Fig. 8b) and differential gene expression analysis revealed incremental downregulation of homeostasis markers throughout the WT, 5×FAD, *Cnp*^*−/−*^ and *Cnp*^*−/−*^;5×FAD trajectory with concomitant increases in activation and inflammatory genes (Fig. 4e,f and Extended Data Fig. 8a–e). Clustering of differentially expressed genes (DEGs) based on normalized expression and subsequent gene ontology (GO) enrichment analysis confirmed an overall more inflammatory profile of microglia isolated from *Cnp*^*−/−*^ mice (Extended Data Fig. 8d,e and Supplementary Tables 1 and 2). Classical DAM signature genes were upregulated in microglia from 5×FAD mice and were further increased in microglia from *Cnp*^*−/−*^ mice and *Cnp*^*−/−*^;5×FAD mice (Fig. 4e,f and Extended Data Fig. 8c). DEG analysis between *Cnp*^*−/−*^;5×FAD mice and 5×FAD mice identified many previously reported DAM signature genes (*Clec7a*, *Gpnmb*, *Apoe*, *Spp1*, *Axl* and *Itgax*) and genes of the *Ms4a* cluster as highly upregulated in *Cnp*^*−/−*^;5×FAD mice (Extended Data Fig. 8f–h). Such a signature was previously described to be a feature of highly phagocytic microglia in early development and for brain border macrophages^38^*. Trem2* and *Tyrobp*, however, showed unaltered induction levels (Fig. 4f). As *Ms4a* genes can regulate the level of soluble TREM2 and modify AD risk^39^, we used immunoblot analysis to rule out the possibility that myelin dysfunction affects TREM2 protein levels or cleavage through increased *Ms4a* expression (Extended Data Fig. 9a). Functional enrichment analysis revealed upregulation of GO terms and pathways related to the immune response in *Cnp*^*−/−*^;5×FAD mice (Extended Data Fig. 8i). STRING (Extended Data Fig. 8i) and DEG analyses showed substantial upregulation of lipid-metabolism-related genes (*Apoe*, *Apoc1*, *Apoc4* and *Lpl*) in *Cnp*^*−/−*^;5×FAD mice (Extended Data Fig. 8j). Results of co-immunostaining experiments showed increased levels of APOE, a well-known factor in Aβ aggregation^40^, in microglia (Fig. 4g) and amyloid plaques in *Cnp*^*−/−*^;5×FAD mice (Fig. 4h).

**Fig. 5:**
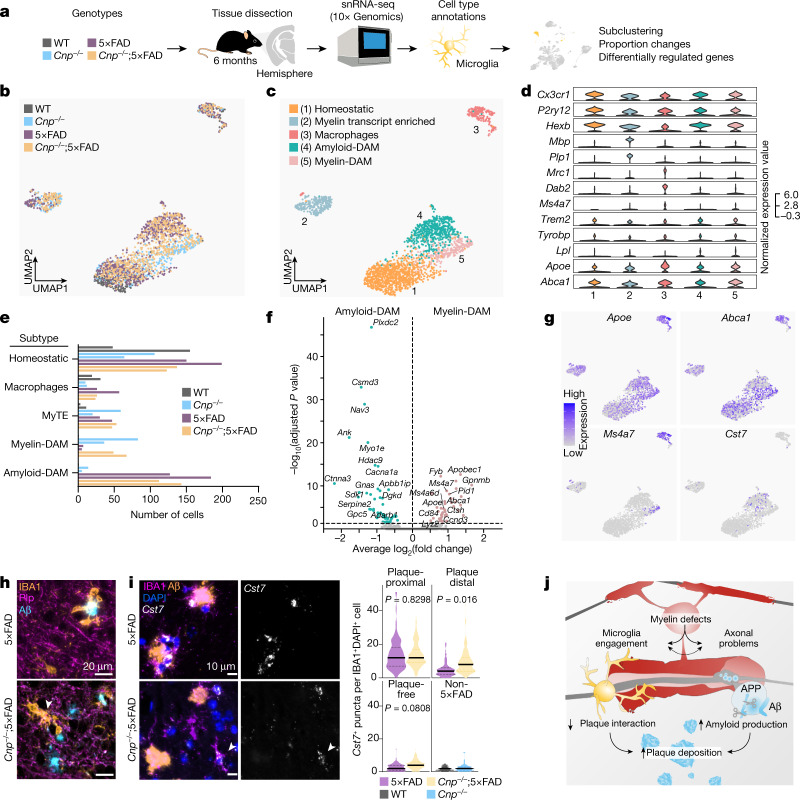
Myelin dysfunction induces a DAM-like state distinct to amyloid deposition as determined by snRNA-seq. **a**, Experimental setup for studying microglia states associated with myelin disease (myelin-DAM) and amyloid-disease (amyloid-DAM) and in combination. Brain hemispheres were isolated from 6-month-old WT, *Cnp*^*−/−*^, 5×FAD and *Cnp*^*−/−*^;5×FAD mice and subjected to snRNA-seq. Cell types were identified on the basis of marker genes, and microglia were subset for further analysis. **b**,**c**, Uniform manifold approximation and projection (UMAP) visualization of microglia subsets coloured by genotypes (**b**) or subpopulations (**c**). **d**, Violin plots showing expression of microglia subpopulation marker genes. **e**, Bar plot showing the distribution of microglia subpopulations across genotypes. MyTE, myelin transcripts enriched. **f**, Differentially regulated genes between amyloid-DAM and myelin-DAM. **g**, Feature plots showing expression of *Apoe*, *Abca1*, *Ms4a7* and *Cst7* in microglia subpopulations. **h**, Representative example images of microglia distraction in *Cnp*^*−/−*^;5×FAD mice. The arrowhead in the bottom panel highlights an activated microglial cell that is engaged in myelin phagocytosis and does not react to nearby plaques. The same observations were made in five independent samples per mice per group. **i**, Left, amyloid and microglia immunostaining and single-molecule fluorescence in situ hybridization for the amyloid-DAM marker *Cst7*. Right, violin plots show the amount of *Cst7* signal per microglia. Microglia were separated into groups according to their location in relation to amyloid plaques (plaque-proximal, plaque-distant and plaque-free regions). The following number of microglia were analysed: 5×FAD: plaque-proximal = 161, plaque-distant = 111, plaque-free = 128; *Cnp*^*−/−*^;5×FAD: plaque-proximal = 136, plaque-distant = 184, plaque-free = 184 from 3 mice per replicates per group (*n* = 3). For *Cnp*^*−/−*^, *n* = 61 and for WT, *n* = 63 from one animal each. Black lines indicate medians. Statistical analysis: two-sided, unpaired Student’s *t*-test (*P* values are indicated in the graphs) on biological replicate data. **j**, Scheme illustrating how myelin dysfunction acts as an AD risk factor. Upstream myelin defects cause microglia engagement and axonal transport problems. The two downstream pathologies are probably interrelated (dashed arrow). Axonal problems lead to endosome and lysosome accumulation and enhanced amyloid production. Simultaneously, microglia become increasingly engaged with defective myelin, which reduces their interaction with amyloid plaques. Both processes contribute to the enhanced deposition of amyloid. Source data

**Extended Data Fig. 10:**
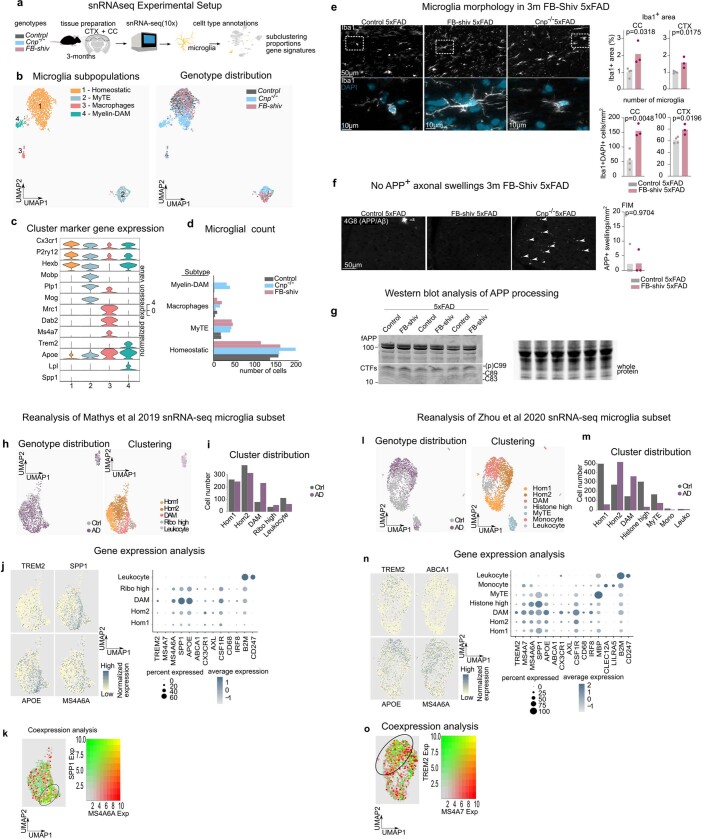
Lack of microglia activation and APP metabolism changes in *forebrain shiverer 5xFAD* mice and MS4A cluster gene expression in human AD microglia. (**a**) Experimental setup to study microglia states in *FB-shiv* (*forebrain shiverer)* in comparison to wildtype controls and *Cnp*^*−/−*^ brain by snRNAseq at 3 months of age. (**b**) Identification of microglia/macrophage subpopulations and genotype distribution visualised in the UMAP space. (**c**) Marker gene expression for the four identified clusters visualised in violin plots. (**d**) Number of microglia per microglia subtype and genotype. Different bars represent single animals/biological replicates (n = 2 per group). (**e**) Assessment of microglia coverage and morphology by Iba1 stainings in 3-month-old *FB shiverer 5xFAD* mice. Lower panel shows closeups of cells delineated by dashed lines in the upper panel. As a comparison and positive control for activated microglia a micrograph of *Cnp*^*−/−*^*5xFAD* is shown on the right. Bargraphs show quantification of Iba1^+^ area and microglia number in the cortex (CTX) and corpus callosum (CC). Bars represent means, dots represent animals/biological repolicates/n (n = 4 for control and n = 3 for *FB shiverer* mice). Statistical analysis: two-sided Student’s t-test (p-value is given in the graph). (**f**) Assessment of APP+ axonal swellings in the fimbria (FIM) of 3-month-old *FB shiverer 5xFAD* by 4G8 (APP/Aβ) immunolabeling. In both control *5xFAD* mice and *FB shiverer 5xFAD* APP+ swellings were rarely or not found. The contoured arrow in the left micrograph highlights an amyloid plaque. As a comparison and positive control for APP+ swellings a micrograph of *Cnp*^*−/−*^*5xFAD* is shown on the right. Swellings are highlighted by arrowheads. Bars represent means, dots represent animals/biological repolicates/n (n = 4 for control and n = 3 for *FB shiverer* mice). Statistical analysis: two-sided Student’s t-test (p-value is given in the graph). (**g**) Western blot analysis of APP processing in *FB shiverer 5xFAD* mice. Bands for full-length APP (fAPP) and c-terminal fragments (CTFs) are shown. n = 3 biological replicates/mice/lanes per group. Source data are given in Supplementary Fig. 1. Molecular weight marker (in kDa) is given on the left. (**h**-**k**) Reanalysis of microglia subcluster of Mathys et al.^46^. (**h**) Genotype and clustering distribution of the microglia/leukocyte subset in the Mathys et al.^46^ snRNA-seq dataset visualised in the UMAP space. Hom = homeostatic microglia, DAM = Disease-associated microglia, Ribo high = high in ribosomal transcripts. (**i**) Histogram representation of cell numbers in each subcluster according to sample condition (control versus AD). Bars represent cell numbers. (**j**) Expression analysis of classical DAM marker genes (*TREM2, SPP1, APOE*) and *MS4A6A* in the main microglial subcluster (leukocytes not shown) visualised in the UMAP space (left) and by dot plot representation (right). (**k**) Co-expression analysis of MS4A6A and SPP1 at the single-cell level visualised in the UMAP space. Colour indicates gene expression and co-expression level according to the colour scale shown on the right. (**l**-**o**) Reanalysis of microglia subcluster of and Zhou et al.^35^. (**l**) Genotype and clustering distribution of the microglia/leukocyte subset in the Zhou et al.^35^ snRNA-seq dataset visualised in the UMAP space. Hom = homeostatic microglia, DAM = Disease-associated microglia, Histone high = high in histone transcript, MyTE = Myelin transcript enriched. (**m**) Histogram representation of cell numbers in each subcluster according to sample condition (control versus AD). (**n**) Expression analysis of classical DAM marker genes (*TREM2, APOE*), *ABCA1*, and *MS4A6A* in the main microglial subcluster (leukocytes, monocytes and MyTE not shown) visualised in the UMAP space (left) and by dot plot representation (right). (**o**) Co-expression analysis of *MS4A7* and *TREM2* at the single-cell level visualised in the UMAP space. Colour indicates gene expression and co-expression level according to the colour scale shown on the right. The results published here are in whole or in part based on data obtained from the AD Knowledge Portal (https://adknowledgeportal.org). Source data

To better understand the DAM-like microglial signature induced by myelin defects, we further performed single-nuclei RNA-seq (snRNA-seq) on brain tissue from *Cnp*^*−/−*^;5×FAD mice and respective controls (Fig. 5a and Extended Data Fig. 9b–d). Cluster analysis identified five major microglia and macrophage subpopulations, including two distinct clusters (clusters 4 and 5) with high expression of DAM marker genes^37^ (*Trem2*, *Lpl* and *Spp1*) (Fig. 5b–d). Cluster 4 was almost exclusively derived from cells with the 5×FAD genotype, whereas cluster 5 consisted of cells from the *Cnp*^*−/−*^ background (Fig. 5e). We therefore termed these two clusters myelin-DAM and amyloid-DAM, respectively. Differential expression analysis between myelin-DAM and amyloid-DAM revealed upregulation of lipid-metabolism-related genes (*Apoe*, *Abca1* and *Apobec1*) and genes of the *Ms4a* cluster in myelin-DAM, which corroborated findings from our bulk RNA-seq experiment (Fig. 5f and Extended Data Fig. 9e). Genes specifically upregulated in amyloid-DAM included *Cst3*, *Ctnna3* (which encodes alpha-T-catenin) and *Gpc5* (which encodes the heparan sulfate proteoglycan glypican 5). Despite a low detection rate, *Cst7* was also specifically enriched in amyloid-DAM (Fig. 5g). Notably, when comparing microglia from *Cnp*^*−/−*^;5×FAD mice with 5×FAD mice within the amyloid-DAM cluster, we still observed upregulation of *Apoe in Cnp*^*−/−*^;5×FAD microglia (Extended Data Fig. 9g,h). Analyses of DEGs shared between DAM clusters and genotype comparisons within DAM clusters revealed that both amyloid-DAM and myelin-DAM of the *Cnp*^*−/−*^;5×FAD crossbred mice retained signature features of the other DAM cluster, which highlights the intermediate phenotype of microglia in this mouse model (Extended Data Fig. 10h,i).

Overall, our single-nucleus transcriptome analysis indicated that microglia respond in a similar but distinct manner to myelin dysfunction and the amyloid plaques they encounter. In the *Cnp*^*−/−*^;5×FAD model, in which the two pathologies co-exist, both microglia states are adequately induced (despite modifications to the signatures). Our imaging data, by contrast, showed an almost complete loss of plaque-corralling microglia, which were previously identified as the in situ equivalent to DAM^37^. We wondered whether myelin damage would engage a large fraction of microglia in myelin clearance, thereby distracting even successfully induced amyloid-DAM away from plaques. Immunostaining for three markers showed that a large number of microglia and plaque-diverted microglia in *Cnp*^*−/−*^;5×FAD mice seemed to be reactively engaged in myelin phagocytosis (Fig. 5h and Extended Data Fig. 9j). *Cst7* in situ hybridization—a marker for amyloid-DAM—showed that amyloid-DAM in *Cnp*^*−/−*^;5×FAD mice were frequently found distal to the plaque, which was in contrast to 5×FAD control mice (Fig. 5i). We propose that such distraction of microglia leads to a faster build-up of amyloid in the brain. Moreover, factors secreted by activated but myelin-engaged microglia (such as APOE) may further fuel plaque seeding.

We next investigated whether altered microglia phenotypes also contribute to amelioration of plaque burden in forebrain shiverer;5×FAD mice (which lack myelin in the forebrain). To this end, we performed snRNA-seq analysis of microglia states in forebrain shiverer mice and contrasted those with the microglia signature found in *Cnp*^*−/−*^ animals at 3 months of age (Extended Data Fig. 10a). In forebrain shiverer mice, we failed to detect activated microglia or myelin-DAM compared with microglia from *Cnp*^*−/−*^ mice (Extended Data Fig. 10b–d). Similarly, immunostaining showed that microglia from forebrain shiverer*;*5×FAD mice remained in an homeostatic state and displayed a highly ramified morphology. However, we detected an increase in microglia cell numbers (Extended Data Fig. 10e). This could convey a certain protection against plaque build-up early in disease as more microglia could potentially clear amyloid. Also, contrary to mice with dysfunctional myelin, the lack of myelin in forebrain shiverer;5×FAD mice did not lead to axonal swellings or alterations in APP metabolism (Extended Data Fig. 10f,g).

Our findings and experimental models (myelin defects on top of an AD background) are highly relevant to the human disease as they mimic the state of the old ‘comorbid’ brain. That is, microglia would see multiple pathological stimuli simultaneously—such as age-related myelin breakdown and amyloid—similar to microglia in *Cnp*^*−/−*^5×FAD mice. A re-analysis of published human AD snRNA-seq datasets showed that activated microglia—in contrast to pure amyloid-DAM in 5×FAD mice—showed upregulation of *MS4A* cluster genes, thereby demonstrating a myelin clearance-related signature (Extended Data Fig. 10h–o).

## Ageing myelin as a risk factor for AD

Only a few studies have experimentally investigated oligodendrocytes and myelin in amyloid mouse models of AD^20,21,41–43^. Nearly all have focused on the effect of amyloid plaques and Aβ on oligodendrocytes and myelin, partially describing secondary demyelination or hypomyelination. Similarly, single cell RNA-seq analysis of mouse models of AD demonstrated signature alterations in oligodendrocytes, specifically nearby amyloid plaques^44,45^. RNA-seq studies using human AD autopsy samples have identified myelin-related transcripts among the top altered gene clusters in patients with AD^35,46^. These findings, however, probably reflect the downstream effects of overt Aβ and tau pathology on oligodendrocytes in the latest stages of disease. In our study, we specifically asked whether myelin defects are upstream of amyloid deposition.

Based on our findings, we propose a working model for AD (Fig. 5j) in which myelin dysfunction in the ageing forebrain (modelled by the premature ageing of white matter in myelin mutant mice) causes microglia to become engaged. This microglial activity interferes with the ability to clear Aβ deposits and prevent plaque formation. Simultaneously, ageing myelin loses its axon-supportive functions, leading to axonal distress, which in turn increases neuronal BACE1 and APP CTF levels suggestive of enhanced Aβ production. It is tempting to speculate that other well-known AD risk factors, such as traumatic brain injury or cardiovascular diseases, also convey risk by negatively affecting myelin health or at least through similar mechanisms (that is, distracting microglia).

The identified plaque-promoting factors, axonal distress and microgliosis are tightly linked downstream phenomena of myelin injury, which makes it difficult to study them in isolation. We have previously shown that depletion of microglia in *Cnp*^*−/−*^ mice can reduce the number of axonal swellings^47^, which indicates that axonal distress and APP metabolism changes could be downstream of microglia activation. However, nonspecific microglia activation induced by lipopolysaccharide does not have the same amyloid-promoting effect as myelin dysfunction, as increases, decreases or no changes in amyloid burden have been reported^48–51^. By contrast, myelin defects seem to be unique drivers of microglia activation in the ageing brain (as previously shown^3^), which challenge microglia with the uptake and digestion of large amounts of lipids and highly compacted membranes.

An important clinical implication of our findings is the co-existence of AD and multiple sclerosis (MS). Based on our findings in EAE and cuprizone-treated AD mice, we would suspect an increased rate of AD as a comorbidity of MS. A recent study^52^ has shown that patients with MS have a higher risk of receiving an AD or dementia diagnosis. However, given the very limited dataset, further epidemiological studies concerning the comorbidity of MS and AD are needed^53^.

A role of myelin deterioration in the progression of AD has been previously proposed in light of correlative macroscopic-scale brain imaging studies^54^, but they lacked any evidence of causality. Our experimental findings in genetic models of AD now provide a causal link with a molecular underpinning and are in line with the prevailing amyloid hypothesis and the role of neuroinflammation in disease progression. Moreover, we can position the well-documented loss of myelin integrity in the ageing primate brain as an upstream initiator of AD pathology, which might help to explain why age is the major AD risk factor. Our working model is further supported by previous classical neuropathological observations^55^, in which a conspicuous temporal relationship with CNS myelination was reported, which occurs latest in those brain regions that are the first to develop AD pathology. Mechanistically, cortical ensheathment by oligodendrocytes is thin and may lack the distinct myelinic channel architecture important for glial metabolic support found in subcortical tracts that appear resistant to AD pathology.

Our discovery that age-dependent loss of myelin integrity can be a driver or risk factor of amyloid deposition changes our view on the role of oligodendrocytes in AD—from passive bystanders to active contributors of disease progression. If further corroborated in humans, promoting myelin health should be considered as a therapeutic target for the delay or prevention of AD.

## Methods

### Human tissue analysis

Selection of patients was performed from a pool of approximately 400 individuals, in which an autopsy with neuropathological evaluation was performed between 2018 and 2019 as a matter of routine procedure following death at the Leipzig University Hospital. Ethics oversight was performed by the Ethics Board of the Leipzig University Hospital. In the individual contracts that govern medical treatment, all patients included in the study provided written consent following hospital admission to the scientific use of tissue removed and stored after any biopsy or during autopsy.

Selection of patients was performed according to exclusion and inclusion criteria. Samples were anonymized and processed in a blinded manner. Selected patients were of mixed age and sex, aged between >60 years and <90 years of age, had a clinical history of dementia and a National Institute on Aging–Alzheimer’s Association (NIA–AA) score in neuropathological assessment between A2B3C2 and A3B2C2 (inclusion criteria) and did not suffer from another severe neurological disorder (exclusion criteria). In addition, individuals of the same age range without any clinical or neuropathological record of neurological disease were selected. No other criteria besides the described characteristics were applied. In total, paraffin-embedded CNS tissue from three patients with moderate to pronounced AD neuropathological changes according to the NIA–AA (see above) and from three unaffected individuals were histologically evaluated. In our analysis, we used biopsy samples from the medial temporal lobe containing the hippocampal formation. We performed histological assessment for intracortical myelin content on the human tissue provided. For this, we sectioned paraffin blocks (5 µm) and stained paraffin sections for CNP, PLP, IBA1 and amyloid plaques simultaneously (see the section ‘Paraffin slices and histological stainings’ for details). Quantification of microgliosis (IBA1) and myelin levels (CNP and PLP) were performed in the trans-entorhinal cortex by performing thresholding, and the percentage of positive area was determined using Fiji in equally sized ROIs in the transentorhinal cortex.

### Mouse strains and husbandry

Animal experiments were conducted in compliance with German animal welfare practices and approved by the local authorities (Landesamt für Verbraucherschutz und Lebensmittelsicherheit, Niedersachsen). Mice were group-housed in the local animal facility of the Max Planck Institute for Multidisciplinary Sciences under a 12-h dark–12-h light cycle and fed ad libitum. Both sexes were used throughout the study. The following original mouse strains were used to generate crossbreeedings: 5×FAD (ref. ^56^), *APP*^*NLGF*^ (ref. ^57^), *Cnp*^*−/−*^ (ref. ^58^), *Plp*^*−/y*^ (ref. ^59^), *Plp*^*fl/fl*^ (ref. ^60^), *Mbp*^*fl/fl*^ (ref. ^61^), *Emx*^*cre*^ (ref. ^62^) and *Foxg1*^*cre*^ (ref. ^63^). For analysis, either littermate controls were used or a corresponding control line from the initial F_1_ generation was generated. The age of animals is given in the respective figure legend. All animals were maintained on a C57BL/6 background. Genotyping was performed on clips derived from earmarking according to standard protocols (see references for original mouse strains). The genotype was confirmed by regenotyping on a tail biopsy following euthanasia of the animal at the end of the respective experiment.

### Demyelination models

As demyelination models, we used cuprizone and EAE, and experiments were conducted as previously described^64^. For cuprizone-mediated demyelination, male 14-week-old 5×FAD mice were fed a powder diet containing 0.2% (w/w) cuprizone (Sigma-Aldrich) for 4 weeks followed by a 4-week recovery period on normal pelleted food without cuprizone supplementation. Control 5×FAD mice received a standard diet without cuprizone. Animals were perfused at 18 weeks of age, and brain tissue was analysed by LSM and epifluorescence microscopy. For EAE experiments, 10-week-old 5×FAD mice were immunized against myelin oligodendrocyte glycoprotein (MOG) by subcutaneously injecting 200 mg MOG(33–35) peptide in complete Freund’s adjuvant (*Mycobacterium tuberculosis* at 3.75 mg ml^–1^; BD) followed by injecting 500 ng of pertussis toxin (Sigma) at day 1 and 3 after EAE induction (d.p.i. 1 and 3). Animals were checked on a daily basis, and a neurological disease score was determined according to the following parameters: 0, normal; 0.5, loss of tail tip tone; 1, loss of tail tone; 1.5, ataxia and mild walking deficits (slip off the grid); 2, mild hind limb weakness, severe gait ataxia and twist of the tail causing rotation of the whole body; 2.5, moderate hind limb weakness and inability to grip the grid with the hind paw but ability to stay on an upright tilted grid; 3, mild paraparesis and falls from an upright tilted grid; 3.5, paraparesis of the hind limbs (legs strongly affected but move clearly); 4, paralysis of the hind limbs and weakness in the forelimbs; 4.5, forelimbs paralysed; 5, moribund or dead. Animals were perfused at 28 d.p.i. at 14 weeks of age. All immunized 5×FAD animals developed EAE. Brain and spinal cords were analysed by LSM and epifluorescence microscopy.

### Mouse behavioural testing

Hindlimb clasping was assessed by suspending the mouse on its tail for 5 s, carefully observing movement of the hindlimbs and scoring movement impairments according to a score from 0–4 as follows: 0, no impairment, hindlimbs normally spread and moving; 1, one hindlimb shows slightly less mobility; 2, both hindlimbs show less mobility; 3, both hindlimbs show reduced mobility and reduced spread; 4, both hindlimbs show severely reduced movement and severely reduced spread. Videos were recorded. Animals were tested in the elevated plus (EPM) and Y-maze (YM) on consecutive days. In the EPM experiment, animals were allowed to freely explore an elevated cross-shaped platform with two opposing enclosed arms and two opposing open arms for 5 min without the experimenter present. In the YM experiment, animals were allowed to explore a Y-shaped maze with enclosed arms for 8 min without human interference. Videos were recorded and analysed using the Bioobserve Viewer behavioural analysis setup with automated tracking. Zones (open arms, closed arms, centre) and object detection settings were optimized according to the maze type used. After the run, positional data, track length and full track images were exported. For the EPM experiment, the time spent in both open arms was summed and plotted. For the YM experiment, the order of arm entries was recorded, and the number of correct triads (consecutive visit of the three different arms) determined. The alternation index was calculated according to the formula alternation index = number trials/number of arm entries – 2. For a successful visit to an arm of the maze, animals had to enter an arm with their full body (excluding the tail).

For statistical analysis of the behavioural data, we report the results of several different type 3 analysis of variance (ANOVA) tests that calculated the main effects for the 5×FAD mice and myelin mutant mice as well as their interaction. All analyses were conducted in R (v.4.04). The ANOVA tests were computed using the afex package (afex v.0.28-1)^65^. Post hoc testing was performed using Tukey’s multiple comparison test in Prism 8.

### Tissue preparation

For microscopy analysis, animals were deeply anaesthetized or euthanised by CO_2_ asphyxiation and subsequently transcardially perfused with ice-cold Hank’s buffered salt solution and 4% paraformaldehyde (PFA) in 0.1 M phosphate buffer, pH 7.4. Brains and spinal cords were removed and post-fixed overnight in 4% PFA and phosphate buffer. Brains were washed once in PBS pH 7.4 and stored in PBS at 4 °C until further use. For biochemical analysis of full brains, animals were killed by cervical dislocation, brain and spinal cord were extracted and fresh-frozen on dry ice. Tissue was stored at −80 °C until further use. For microdissection of subcortical white matter and cortical tissue, animals were killed by cervical dislocation and the brain was quickly removed and submerged in ice-cold Hank’s buffered salt solution. The brain was inserted into a custom-made brain matrix and the brain was sliced coronally in about 1-mm-thick slices. Brain slices were spread onto an ice-cold glass plate, and cortex and subcortical white matter were separated and excised from each individual brain slices. Tissue was immediately frozen on dry ice and stored at −80 °C until further use.

### Whole tissue staining and clearing for LSM

Fixed hemibrains and spinal cords were pretreated and permeabilized following a modified iDISCO protocol^66^. In brief, tissue samples were dehydrated with an ascending concentration of methanol in PBS (50% once, 80% once, 100% twice, 1 h each). Tissue samples were then bleached with a 1:1:4 ratio of H_2_O_2_:DMSO:methanol overnight at 4 °C. Further dehydration was followed by 100% methanol incubation at 4 °C (30 min), −20 °C (3 h) and overnight storage at 4 °C. Samples were incubated the following day in methanol with 20% DMSO before subjecting them to gradual rehydration in a descending methanol in PBS series (80% once, 50% once, 0% once, 1 h each). We then washed the tissue samples with a detergent mixture of 0.2% Triton X-100 in PBS (twice, 1 h) and permeabilized them overnight at 37 °C in PBS with 0.2% Triton X-100, 20% DMSO and 0.3 M glycine. After permeabilization, tissue samples were either stained with the Congo red dye (Sigma Aldrich) for β-sheet structures within amyloid plaques or immunolabelled with antibodies of interest. For Congo red staining, tissue samples were washed with PBS and 0.2% Tween-20, 10 mg ml^–1^ heparin, 5 mM sodium azide (PTwH; twice, 1 h) before immersing them for 3 days at 37 °C in 0.005% w/v Congo red (100× stock solution in 50% ethanol). For immunolabelling, following tissue permeabilization and glycine treatment, samples were blocked in PBS with 0.2% Triton X-100, 10% DMSO and 6% goat serum (GS) for 3 days followed by washing in PTwH (twice, 1 h) and incubation in primary antibodies with the appropriate dilution factors (1:500, rabbit, anti-IBA1, Wako) in PTwH with 0.2% Triton X-100, 5% DMSO and 3% GS for 14 days at 37 °C. Following completion of labelling with primary antibody, tissue samples were washed in PTwH (6 times, 10 min) and stored overnight at 37 °C. For secondary antibody labelling, tissue samples were again incubated in secondary antibodies with the appropriate dilution factors (anti-rabbit Dylight 633, 1:500, Thermo-Fisher) in PTwH with 3% GS for 7 days at 37 °C. Before clearing, spinal cords were fixed in 1.5% w/v Phytagel in water. Dyed or immunolabelled tissue samples were washed in PTwH (3 times, 10 min each) before rehydration through an ascending concentration of methanol in PBS (20% once, 40% once, 60% once, 80% once, 100% once, 1 h each) and delipidation in a 1:2 mixture of methanol:dichloromethane (once, 1 h 40 min). Last, samples were cleared by immersing them in ethyl cinnamate (Eci, Sigma-Aldrich) until transparent. All incubation steps were carried out at constant medium-speed rotation at the indicated temperatures. Samples were stored at room temperature in Eci until imaging.

### In toto imaging of whole tissues and analysis and visualization

Cleared hemibrains and spinal cords were imaged in toto with a light sheet microscope setup (UltraMicroscope II, LaVision Biotec) equipped with a ×2 objective lens, a zoom body and a corrected dipping cap. Samples were imaged submerged in a sample chamber containing Eci. For all hemibrain imaging, hemibrains were mounted medial-side down on the sample holder to acquire sagittal images. Images were acquired using ImspectorPro (v.7.124, LaVision Biotech) software in the mosaic acquisition mode with the following settings: 5 µm light sheet thickness, 20% sheet width, 0.154 sheet numerical aperture, 4 µm *z*-step size, 1,000 × 1,600 pixels field of view, 4 × 4 tiling, dual light sheet illumination and 100 ms camera exposure time. Red fluorescence was recorded with 561 nm laser excitation at 80% laser power and a 585/40 nm emission filter. Far-red fluorescence was recorded with 640 nm laser excitation at 30% laser power and a 680/30 nm emission filter. Autofluorescence was recorded with 488 nm laser excitation at 50% and 525/20 nm emission filter. Images were imported into Vision4D (v.3.2; Arivis) and stitched using the tile sorter setup. For some, images were imported and stitched using Imaris Importer and Stitcher (v.9.1; Bitplane). Rendered whole hemibrains were then processed, and three main ROIs were manually annotated based on the sagittal Allen mouse brain atlas, namely the isocortex, the hippocampus and the alveus. All ROIs were first manually traced in 2D planes to automatically extrapolate the 3D ROIs. Cortical and hippocampal annotations were cropped with a medial cutoff of approximately 0.4 mm and a lateral cutoff of 4.4 mm, which would span the dorsal isocortex and the entire hippocampal formation of one hemibrain. The lateral cutoff for the alveus ROI is the plane where the hippocampal formation appears in 2D. Next, we segmented amyloid plaques within the ROIs. For 3-month-old 5×FAD brain data, we typically used automated intensity thresholding. For 6-month-old 5×FAD data, plaques were segmented using the blob finder algorithm in Vision4D with the following parameters: 20 µm object size, 10–15% probability threshold and 0% split sensitivity. Once segmentation was performed, stringent noise removal was performed by deleting objects with voxel sizes <10 from the object table. Objects were then carefully reviewed, and any additional noise, which might include but are not restricted to blood vessels and nuclei, were manually removed from the dataset. For quantification of amyloid plaques in the *APP*^*NLGF*^ brains that typically stained much weaker for Congo red, plaque segmentation was performed using the machine-learning segmenter in Vision4D. Object parameters and ROI volumes were extracted for further quantification. For plaque visualization in the different ROIs, objects are represented in centroids and colour-coded according to location. The following different plaque burden parameters were assessed: plaque count (number of plaques in ROI), total plaque volume (sum of all individual plaque volumes), plaque size (average volume of plaques), plaque density (number of plaques normalized to ROI volume), percentage volume (ROI volume covered by sum of plaque volume).

### Paraffin slices and histological stainings

Fixated hemibrains and spinal cords were subjected to dehydration steps (50% ethanol, 80% ethanol, 100% ethanol, 100% isopropanol, 50% isopropanol and 50% xylol, twice 100% xylol) followed by paraffinization on a STP 120 tissue processing machine (Leica Microsystems). Samples were retrieved and embedded in paraffin blocks on a HistoStar embedding workstation (Epredia). Paraffin-embedded blocks were sectioned coronally, whereas spinal cords were sectioned longitudinally at 5-μm slice thickness, slices were mounted onto slides and dried overnight. Slides were deparaffinized at 60 °C followed by incubation in xylol (100% twice) and a 1:1 mixture of xylol and isopropanol (once) for 10 min each. The slides were rehydrated in a descending ethanol series. This was followed by incubation in either acidic antigen retrieval solution (pH 6.0) or basic antigen retrieval solution (10 mM Tris and 1 mM EDTA pH 10) for 5 min and boiling for 10 min. The samples were cooled for 20 min and washed in distilled water for 1 min before subsequent permeabilization in 0.1% Triton X-100 in PBS. For Aβ antibody staining, samples were subjected to an extra antigen retrieval step in 88% formic acid to loosen the β-sheet structure for optimal antibody binding for 3 min. For plaque ApoE staining, formic acid treatment was prolonged (10 min, fresh formic acid). This was followed by washes in PBS (twice, 5 min) and blocking with 10% GS in PBS for 1 h at room temperature. For chromogenic labelling, an additional step of inactivation of endogenous peroxidases was implemented before blocking by incubation in 3% H_2_O_2_. After blocking, slices were incubated in primary antibody solution (PBS, 10% GS or PBS, 5% BSA) overnight at 4 °C in coverplates (Epredia). The following antibodies were used in this study: anti-IBA1 (rabbit, Wako; 1:1,000); anti-Aβ-6E10 (mouse, BioLegend; 1:1,000); anti-CNP (mouse, AMAb91072, Atlas; 1:1,000); anti-PLP-clone aa3 (rat, culture supernatant; 1:200); anti-BACE1 (rabbit, ab183612, Abcam; 1:100); anti-MBP (rabbit, serum, custom-made in the Nave Laboratory; 1:1,000); anti-GFAP (mouse, GA5, Leica; 1:200); anti-N-terminal APP (22c11, Merck; 1:1,000); anti-C-terminal APP (rabbit, 127-003, Synaptic Systems; 1:1,000); anti-Aβ_*x*–42_-D3E10 (rabbit, Cell Signaling Technology; 1:1,000); anti-PSEN2 (rabbit, Abcam; 1:100); anti-ApoE D7I9N (rabbit, Cell Signaling Technology; 1:500); anti-ApoE clone 26c11 (mouse, 1:500, provided by C. Haass Laboratory); sAPPβ_swe_ clone 6A1 (mouse, IBL; 1:1,000); anti-Aβ_1–*x*_ (mouse, 80C2, Synaptic Signalling; 1:200); anti-Aβ_1–*x*_ (mouse, Moab2-6C3, Abcam; 1:200); anti-Aβ_4–*x*_ (guinea pig, 029-2 (ref. ^67^), custom-made by O.W.; 1:200); and anti-APP/Aβ_17–24_ clone 4G8 (mouse, BioLegend; 1:1,000). For immunofluorescence staining, samples were washed in PBS or Tris and 2% milk and incubated with the corresponding fluorescent secondary antibody diluted in PBS containing 10% GS for 2 h at room temperature in the dark. The following fluorescently conjugated secondary antibodies were used: anti-mouse Alexa555 (donkey/goat, Thermo-Fisher; 1:1,000); anti-mouse Dylight633 (donkey/goat, Thermo-Fisher; 1:1,000); anti-rabbit Alexa555 (donkey/goat, Thermo-Fisher; 1:1,000); and anti-rabbit Dylight633 (donkey/goat, Thermo-Fisher; 1:1,000). Amyloid plaques were stained using the β-sheet dye methoxy-X04 (4 μg ml^–1^ in 50% ethanol, Tocris) for 30 min at room temperature and contrasting in 50% ethanol. Nuclei were stained with either DAPI (300 nM, Thermo-Fisher) or ToPro3 (1:1,000, Thermo-Fisher) in PBS for 5 min at room temperature. Slides were again washed in PBS and mounted with Aqua PolyMount mounting medium (PolySciences). For chromogenic labelling, a LSAB2 kit (Dako) for rabbit/mouse and a DAB-Kit from Zytomed was used according to the manufacturers’ protocols. Slides were then rinsed in water, dehydrated and mounted using Eukitt (Sigma-Aldrich).

### In situ hybridization

We performed RNAscope Fluorescent Multiplex assays (ACD Bio) according to the manufacturer’s instructions for both paraffin-embedded tissue and cryo-tissue sections. In brief, paraffin-fixed slices underwent pretreatment consisting of deparaffinization steps at 60 °C for 1 h, followed by incubation in 100% xylol (5 min, twice) and 100% ethanol (2 min, twice). Slides were then incubated with hydrogen peroxide at 40 °C for 15 min. After a wash with dH_2_O, slides were boiled in the target retrieval reagent for 10 min and washed with dH_2_O (15 s, once) and with 100% ethanol (3 min, once). After drying, a hydrophobic barrier was drawn and protease digestion was performed by incubating slides with RNAscope Protease Plus for 15 min at 40 °C. Before applying the probes, slides were washed with dH_2_O (2 min, twice). The following RNAscope probes were used: Mm-CST7-C3 (498711-C3) and Mm-Ms4a7-C2 (314601-C2). The probe was hybridized for 2 h at 40 °C. Slides were stored overnight in a 5×SSC buffer. The following day, signal amplification was performed by applying amplification reagents provided in the kit. This was followed by incubation with HRP corresponding to the channel probe for 15 min at 40 °C and visualization was performed using the OpalTM 690 fluorophore for 30 min at 40 °C. After the final wash, we proceeded with immunohistochemical co-staining. Blocking of the slides was done with 10% GS in PBS for 1 h at room temperature. After blocking, incubation in primary antibody solution (PBS and 10% GS) was done overnight at 4 °C. The following primary antibodies were used: anti-IBA1 (rabbit, Wako; 1:1,000) and anti-Aβ-6E10 (mouse, BioLegend; 1:1,000). *Ms4a7* RNAscope was performed on cryo-sections with IBA1 co-labelling. *Cst7* RNAscope was performed on paraffin sections with 6E10 and IBA1 co-labelling. Slides were washed in PBS (5 min, 3 times) and labelled with corresponding fluorescent secondary antibody solution (PBS and 10% GS) for 2 h at room temperature. The following fluorescently conjugated secondary antibodies were used: anti-mouse Dylight488 (donkey, Thermo-Fisher; 1:1,000) and anti-rabbit Dylight555 (donkey, Thermo-Fisher; 1:1,000). Finally, slides were washed in PBS (5 min, 3 times) and mounted with Aqua PolyMount mounting medium (PolySciences). Imaging was performed using a Zeiss Observer Z1 microscope equipped with Plan-Apochromat ×20/08 objective. Quantification of *Cst7* RNAscope was performed by identifying plaque-corralling microglia and plaque-distant microglia (without obvious plaque interaction), and the amount of *Cst7* puncta within these microglia was counted.

### Epifluorescence and brightfield microscopy

Epifluorescence microscopy was performed on a Zeiss Observer Z1 microscope equipped with Plan-Apochromat ×20/08 and Fluar ×2.5/0.12 objectives, a Colibri 5 LED light source (630 nm, 555 nm, 475 nm and 385 nm excitation), 96 HE BFP, 90 HE DAPI, GFP, Cy3, Cy5, 38 GFP, 43 DsRed, 50 Cy5 Zeiss filter sets, a Axiocam MrM and a SMC900 motorized stage. For whole-brain-slice microscopy, a preview scan at ×2.5 magnification was taken, and focus support points were distributed and manually set for imaging at ×20 magnification in ZEN imaging software (ZEN 2011 v.2.0, Zeiss). Tiled images were stitched in ZEN. For visualization, pseudocolours (cyan, magenta, yellow) were assigned to different channels, the intensity was adjusted and images were exported into ZEN. Brightfield microscopy of DAB-stained slices was performed on a Zeiss Axiophot Imager.Z1 equipped with a Achroplan ×4/0.1, PlanFluar ×20/0.75 and Plan Neofluar ×40/0.75 objectives and a AxioCamMrc camera. For whole-brain-slice microscopy, a preview scan at ×4 magnification was taken, and focus support points were distributed and manually set for imaging at ×40 magnification in ZEN imaging software (Zeiss). Tiled images were stitched in ZEN. Brightness and contrast of RGB images were adjusted and images exported into ZEN.

### 2D microscopy quantification

2D image analysis was performed in Fiji (v.1.53c)^68^ and in ZEN. For quantification of amyloid load, thresholding was performed to segment Aβ deposits (either stained by Me-04 or Aβ plaques), and the positive area was calculated in the respective ROI. For analysis of the plaque-corralling phenotype in the 5×FAD model, in IBA1^+^Aβ^+^ co-stainings, individual plaques were segmented using thresholding, and the IBA1^+^ area in each individual plaque was calculated using a Fiji macro-script. IBA1 coverage was expressed as a percentage. As plaques in the *APP*^*NLGF*^ line are not as compacted as plaques in the 5×FAD model and therefore commonly appear ‘fragmented’, the same type of analysis could not be reliably performed in *APP*^*NLGF*^ mice (overestimation of single plaque structures). For both 5×FAD mice and *APP*^*NLGF*^ mice, the number of plaque-associated microglia per plaque (50 representative plaques with diameter >20 µm for each animal) was manually counted. For quantification of ApoE levels in plaques, ApoE^+^ plaques were segmented, and the raw mean fluorescence per plaque was calculated. For quantification of microgliosis in *Cnp*^*−/−*^;5×FAD, *Plp*^*−/y*^;5×FAD and *Emx*^*cre*^*Mbp*^*fl/fl*^ experimental cohorts, the amount of IBA1^+^DAPI^+^ cells in the ROI (corpus callosum or cortex) was counted and normalized to the area examined. Additionally, the IBA1^+^ area fraction was determined in Fiji by performing thresholding. The number of axonal swellings positive for APP-processing, APP and Aβ antibodies was determined by counting these structures in the respective ROI (fimbria) and normalization to the area examined.

### EM analyses

Fixed brain tissues were immersed in ice-cold PBS and cut into a coronal section with 300 µm thickness using a vibratome (Leica VT1200). Tissue containing the medial corpus callosum and cingulate cortex with adjacent tissue was punched with a 2 mm diameter punching tool and embedded in Epon using a standard Epon-embedding protocol including 4 h of incubation in 2% OsO_4_ (EM TP, Leica). Samples were placed on a Parafilm-covered plate and covered with an Epon-filled gelatin capsule. Polymerization was performed at 60 °C for 24 h. Samples were trimmed, and semi-thin 500 nm sections were cut on a Leica UC7 ultramicrotome (Leica) equipped with a Histo diamond knife (Diatome). Semi-thin sections were stained with Azure II for 1 min to stain lipid-rich areas to confirm the ROI. Next, 60 nm ultrathin sections were cut on the same ultramicrotome equipped with Diatome diamond knife, ultra 35°. Ultrathin sections were transferred to Formvar-coated copper mesh grids (Science Services) and air dried. The sections were contrasted with Uranyless for 30 min (Electron Microscopy Sciences) and washed five times with ddH_2_O. Samples were air dried before further storage. At least 10 images for each area of interest (magnification of ×4,000 for corpus callosum, ×7,000 for cortex) of ultrathin sections were acquired using a Zeiss EM900. Non-overlapping transmission EM micrographs were used to quantify the numbers of myelinated axons and *g*-ratios. Analyses of the images were done using Fiji. The number of myelinated axons was quantified using the cell counter plugin by counting all myelinated axons in the field of view of five pictures for each animal. For *g*-ratio analysis, at least 150 fibres per animal were randomly selected. The area of the axon and the entire myelinated fibre were measured and used to calculate the diameter. To determine the *g*-ratio, the axonal diameter was divided by the diameter of the myelinated fibre. In the results, the *g*-ratio is plotted against the axon calibre.

### High-pressure freezing EM

Sample preparation by high-pressure freezing and freeze substitution was performed as previously described^69^. In brief, optic nerves from 6-month-old *Cnp*^*−/−*^ mice and control WT mice were freshly dissected, immersed in 20% PVP in PBS and placed into HPF sample carriers (Wohlwend). After freezing using a HPM100 high-pressure freezer (Leica Microsystems), samples were embedded in Epon after freeze substitution using 0.1% tannic acid in acetone followed by 2% OsO_4_ and 0.1% uranyl acetate in acetone. After polymerization, samples were sectioned using a UC7 ultramicrotome (Leica Microsystems) and imaged with a LEO912 transmission electron microscope (Carl Zeiss Microscopy) using an on-axis 2k CCD camera (TRS).

### Cell fractionation

For analysis of APP and TREM2 fragmentation, cell fractioning was performed before western blot analysis according to published protocols^36^. In brief, tissue was homogenized in DEA-buffer (0.25% diethylamine and 50 mM NaCl pH 10) using the Precellys bead-milling method (Precellys soft tissue homogenizing lysis kit, Bertin Instruments), and the soluble protein fraction was extracted by centrifugation (10 min, 500*g*, 4 °C) followed by ultracentrifugation (1 h, 130,000*g*, 4 °C). The membrane-bound fraction was solubilized in RIPA buffer (20 mM Tris-HCl pH 7.5, 150 mM NaCl, 1% NP-40, 1% SDS, 2.5 mM sodium pyrophosphate and 1 mM Na_2_EDTA) and cleaned by centrifugation (10 min, 500*g*, 4 °C) and ultracentrifugation (1 h, 130,000*g*, 4 °C). RIPA-insoluble material (containing plaque Aβ) was resuspended in ice-cold 70% formic acid in water, sonicated and ultracentrifuged (1 h, 130,000*g*, 4 °C). Supernatant was collected as the formic acid fraction and neutralized with 1 M Tris pH 9.5. All buffers and solutions were supplemented with protease inhibitor cocktail (P8340, Merck). Fractions were stored at −80 °C until further use.

### SDS–PAGE and western blotting

To determine the protein concentration in samples, detergent-compatible protein assays (Bio-Rad) were carried out in duplicate. Samples were mixed with Laemmli sample buffer (2% SDS, 10% glycerol, 0.0025% bromphenol blue, 0.125 M Tris-Cl, pH 6.8 and 0.05 M DTT) and an equal amount of protein (typically 20–30 µg) was loaded per lane. For BACE1 western blotting, standard Tris-glycine SDS–PAGE gels (8%) were used. For western blot analysis of APP and TREM2 fragmentation, Tris-tricine SDS–PAGE gels (10–20%, Novex, Thermo Fisher) were used. Gels were run at 100–120 V for approximately 1 h. For Tris-glycine SDS–PAGE gels, proteins were transferred onto low-fluorescent Immobilon-FL membrane (0.45 µm pore size, Merck) using the Bio-Rad wet-blot system (1.15 h, 500 mA) and blot transfer buffer (25 mM Tris and 190 mM glycine) containing 20% methanol. For Tris-tricine SDS–PAGE gels, proteins were transferred onto a low-fluorescent PVDF membrane of lower pore size (0.2 µm). Blots were washed in water, and transferred protein was stained with Fastgreen for transfer quality-check and for normalization purposes. For this, membranes were transferred to Fastgreen working solution (0.0005% Fastgreen FCF (Serva)) in de-staining solution (30% methanol, 7% ml glacial acetic acid in 63% H_2_O) for 5 min and briefly washed two times in de-staining solution. Membranes were imaged using a ChemoStar fluorescent imager (Intas) equipped with a 670 nm/20 nm excitation filter and near-infrared emission collection. Membranes were rinsed in TBS with Tween (0.05%) until the pH was neutral and blocked in 5% BSA in TBS-T for 1 h at room temperature. Membranes were then incubated in primary antibodies in 5% BSA overnight at 4 °C on a rotating shaker. The following primary antibodies were used: anti-BACE1 (1:1,000, rabbit, D10E5, Cell Signaling Technologies); anti-C-terminal APP (1:1000; rabbit, A8717, Merck); anti-C-terminal APP (1:1,000; rabbit, 127-003, Synaptic Systems); anti-APP/Aβ (1:1,000; mouse, 6E10, BioLegend); anti-C-terminal TREM2 (1:1,000; rabbit, E7P8J, Cell Signaling Technologies); anti-CNP (mouse, AMAb91072, Atlas; 1:1,000); and anti-MBP (rabbit, serum, custom-made in the Nave Laboratory; 1:1,000). Membranes were washed several times, and membranes were incubated in secondary antibody solution (5% BSA in TBS-T). The following secondary antibodies were used: anti-rabbit IgG (H+L) DyLight 800 (1:1,000; Thermo Fisher) and anti-mouse IgG (H+L) Dylight 680 (1:1,000; Thermo Fisher). Membranes were scanned using an Odyssey platform (Licor). For visualization, scanned images were adjusted for brightness and contrast for optimal display. For quantification (performed on raw images), background was subtracted and bands were analysed using Fiji (integrated density). Protein levels were normalized to Fastgreen whole protein or to full-length protein in the case of APP.

### MACS of microglia and bulk RNA-seq

Microglia were isolated from mouse hemibrains (excluding cerebellum and olfactory bulb) by MACS. Dissected tissues were enzymatically and mechanically dissociated using a Miltenyi Biotec adult brain dissociation kit according to manufacturer’s protocol. Before microglial isolation using CD11b microglia microbeads and LS columns (Miltenyi Biotec), astrocytes (ACSA-2 microbeads) and oligodendrocytes (O4 microbeads) were removed to enhance the purity of the microglial population. Isolated microglia were directly eluted into RLT lysis buffer, and RNA was isolated using a RNeasy Micro kit (Qiagen). In total, *n* = 4 replicates were used for each genotype (WT, *Cnp*^*−/−*^, 5×FAD and *Cnp*^*−/−*^;5×FAD). RNA extracted from sorted mouse brain hemisphere microglia was eluted in 30 µl nuclease-free water and subjected to 50 bp single-end mRNA sequencing using HiSeq 4000 (Illumina). Raw sequencing data were first evaluated using FASTQC (v.0.72) for quality, then aligned against the reference mouse genome GRCm38 using STAR (v.2.5.2b-2)^70^ with default parameters. Gene raw counts of each sample were extracted using featureCounts (v.1.6.3)^71^ from aligned profiles for differential gene expression analysis using DESeq2 (v.1.26.0)^72^ and converted to TPM value for sample distance calculation and visualization, as well as for gene expression pattern analysis. For differential gene expression analysis, each pair of genotype was calculated separately, and statistics results were summarized (Supplementary Table 1). Gene targets with adjusted *P* value < 0.05 were considered as significantly regulated. Using normalized TPM profiles, samples were embedded by PCA to assess distances.

For significantly regulated genes between each pair of genotype, functional and pathway enrichment analysis using over-representation analysis approach was performed using R (v.3.6.3) with the gprofiler2 package (v.0.2.0)^73^, in which gene set enrichment analysis was performed using the WebGestalt interface^74^. DEGs obtained from all paired comparisons were aggregated and clustered based on their expression patterns across genotypes, whereby clusters were identified using *k*-means (*k* = 10) and expression levels were represented by scaled TPM values. GO (biological process) enrichment analysis was performed for each DEG cluster using gprofiler2, and significant terms enriched from all clusters were input for similarity analysis using simplifyEnrichment package (v.1.5.2)^75^ built under R (v4.1.0). To view potentially gene–gene networks of significantly regulated targets (adjusted *P* < 0.01) between *Cnp*^*−/−*^;5×FAD mice and 5×FAD mice, STRING (v.11.5) analysis was applied with the highest confidence (0.900) of interaction score.

### In vitro phagocytosis assay

An Aβ phagocytosis assay was performed on bone-marrow derived macrophages (BMDMs) to assess phagocytosis capabilities of phagocytes after myelin pretreatment. BMDMs were isolated and cultured as previously described^64^. For experiments, cells were detached by accutase treatment and seeded onto poly(l-lysine)-coated, HCl washed 12-mm-diameter glass coverslips (density of 18,000 cells per coverslip). Cells were left untreated (control) or treated with purified myelin membranes (purification by sucrose-gradient ultracentrifugation) at 2.5 µg ml^–1^ for 12 h. Cells were subsequently treated with HiLyte488-labelled aggregated, synthetic Aβ_42_ (Anaspec, AS-60479-01) at 1 µg µl^–1^ for 4 h. Aggregation of Aβ_42_ was induced by previous incubation of Aβ_42_ at 100 µg ml^–1^ in DMEM at 37 °C for 24 h after vortexing the solution for 15 s. Cells were fixed in 4% PFA and PBS and subjected to fluorescence labelling with DAPI and Lectin-Dylight 649 (Lycopersicon Esculentum Tomato; Fisher Scientific, NC1093063). Lectin-Dylight 649 was incubated for 2 h at room temperature in PBS. Optical sections were acquired with a confocal laser-scanning microscope (Zeiss LSM 900 AiryScan) using a LD C-Apochromat ×40/1.1 water objective (zoom factor of 1.0). The *z*-step in *z*-stacks was kept at 0.8 μm. Images were acquired in bidirectional mode (Aryscan Mode SR:4.1 2D Auto) and the LSM scan speed was kept at 7. Laser intensity was kept at 2% 640 nm, 0.1% 561 nm, 0.5% 488 nm and 0.2% 405 nm. Image resolution was selected to be 1,000 × 1,000 pixels at 16 bit. Acquired images were maximum-intensity projected along the *z*-axis, and the amount of phagocytosed Aβ_42_ was determined by thresholding in the respective channel using Fiji. This percentage positive area was normalized to the area covered by cells (as determined by lectin labelling). Two to three images per treated coverslip were analysed.

### Isolation of nuclei and single-nuclei transcriptome sequencing

We generated two snRNA-seq datasets for the analysis of microglia states induced by myelin dysfunction and amyloidosis. For the first dataset, cortex and corpus callosum from 3-month-old WT, forebrain shiverer mice (*Foxg1*^*cre*^*Mbp*^*fl/fl*^), *Cnp*^*−/−*^ mice and WT controls were microdissected, and tissues collected from two animals were pooled for each replicate. For the second dataset, brain hemispheres (without cerebellum and olfactory bulb) were extracted from 6-month-old WT, *Cnp*^*−/−*^, 5×FAD and *Cnp*^*−/−*^;5×FAD mice. Two replicates per genotype were sequenced. Nuclei were isolated according to previously published methods^76^. In brief, frozen tissue was transferred into 2 ml of pre-chilled homogenization buffer (320 mM sucrose, 0.1% NP40, 0.1 mM EDTA, 5 mM CaCl_2_, 3 mM Mg(Ac)_2_, 10 mM pH 7.8 Tris, 167 µM β-mercaptoethanol and 1× protease inhibitor (Roche)). Tissue was carefully homogenized and filtered through a 80 µm strainer and further centrifuged for 1 min at 100 .r.c.f. For each sample, 400 µl supernatant was collected into a pre-chilled 2 ml low-binding Eppendorf tube, followed by adding 400 µl 50% iodixanol solution (in 1× homogenization buffer containing 480 mM sucrose) to reach a 25% iodixanol concentration. By layering 600 µl of 29% iodixanol underneath the 25% iodixanol mixture, then 600 µl of 35% iodixanol underneath the 29% iodixanol layer, two clear interfaces between different concentrations of buffers were created, and the tube was centrifuged for 20 min at 3,000 r.c.f. After centrifugation, nuclei were collected from the band between the 29% and the 35% iodixanol layers and transferred to a fresh pre-chilled tube. Isolated nuclei were washed and resuspended in cold resuspension buffer (1× PBA, 1% BSA and 0.1 U µl^–1^ RNase inhibitor) and further subjected to single-nuclei transcriptome libraries using a chromium single cell 3′ reagent kit according to the manufacturer’s instructions (10x Genomics). The constructed libraries were sequenced using Novaseq 6000 (Illumina). Raw snRNA-seq data were collected in Fastq format. Data generated from the 3-month time point was aligned to reference genome premRNA using CellRanger toolkit (v.3.0.2; 10x Genomics). The 6-month time point snRNA-seq data were first aligned to the reference mm10 genome version index from 10x Genomics (refdata-gex-mm10-2020-A) using CellRanger toolkit (v.6.1.2; 10x Genomics), and aligned profiles were further processed using CellBender (v.0.2.0) for removal of ambient transcripts.

Matrices containing a unique molecular identifier (UMI) count of each gene in each nuclei were extracted for all samples by filtering out nuclei with <200 detected genes and <500 total transcripts, as well as nuclei with outlier level transcript quantity or a gene detection rate identified according to individual sample sequencing depth (Supplementary Table 1). Genes expressed in fewer than three cells were excluded from further analysis. Filtered expression matrices were combined based on data time point (3-month and 6-month), and the UMI of each nucleus were normalized towards its total UMI count with a scale factor of 10,000 and then log transformed.

### Dimensionality reduction, clustering analysis and cell type annotation

The normalized UMI matrix for 3-month and 6-month time point data were mainly analysed using the R package Seurat (v.4.1.1)^77,78^. Highly variable genes were calculated and scaled to support linear dimensionality reduction using PCA. For all cells sequenced from 3-month-old animals, the first 50 PCs were used for further neighbouring embedding using UMAP^79^, as well as for performing the clustering analysis with a resolution of 0.5 using *K*-nearest neighbour algorithm. Cells sequenced from 6-month-old animals underwent the same neighbouring embedding protocol using the first 50 PCs and proceeded with clustering analysis with a resolution of 0.8. Cluster marker genes in both datasets were calculated using both default Wilcoxon test and the MAST algorithm^80^ to carefully determine cluster cell-type annotations. Clusters with undefined cell identities were removed from further analysis. To perform cell-type-specific analysis for microglia, the corresponding cell population from each dataset was first subset and reduced for its dimensionality using PCA. Similarly, selected top PCs were used for UMAP embedding and clustering analysis, with cluster marker genes calculated using the default Wilcoxon test. Specific parameters used for analysing microglia can be found in Supplementary Table 1.

### External human snRNA-seq data microglia subset re-analysis

Previously published human snRNA-seq data—snRNAseqAD_TREM2 (ref. ^35^) and snRNAseqPFC_BA10 (ref. ^46^)—were obtained from AD Knowledge Portal (https://adknowledgeportal.org) following application for data access. Aligned data profiles were downloaded and pre-processed with quality control and removal of outlier cells, referring to parameters that were used in the respective original publications. Cells from each dataset were analysed using Seurat (v.4.1.2). Microglia and macrophage cell populations were identified by a set of marker genes including *CX3CR1*, *P2RY12*, *TMEM119*, *HEXB*, *TREM2* and *SPP1* and further extracted for subset analysis. Microglia subset expression matrices from both datasets underwent data normalization, high variable gene calculation, linear reduction of dimensionality and eventually neighbouring embedding using UMAP. Unbiased clustering analysis was performed at a resolution of 0.5 in both subsets. Cluster marker genes were calculated using default setups compiled in the Seurat analysis pipeline. Subpopulations that were identified as doublets, low-quality cells or contaminations were removed from further analysis and visualizations.

### Data visualization

Images were exported from the respective imaging or bioinformatics software (ZEN 2011 blue edition, Zeiss; Vision4D Arivis; Fiji or R) and final figures were assembled in Inkscape (v.1.1, https://www.inkscape.org). Graphs were created using Prism 8.0 (GraphPad).

### Reporting summary

Further information on research design is available in the Nature Portfolio Reporting Summary linked to this article.

## Online content

Any methods, additional references, Nature Portfolio reporting summaries, source data, extended data, supplementary information, acknowledgements, peer review information; details of author contributions and competing interests; and statements of data and code availability are available at 10.1038/s41586-023-06120-6.

### Supplementary information

Supplementary Information Supplementary Guide and Supplementary Figs. 1–3.

Reporting Summary

Supplementary Table 1

Supplementary Table 2

### Source data

Source Data Figs. 2–5 and Source Data Extended Data Figs. 1–10.

## Data Availability

All raw sequencing data and raw and processed count matrices have been uploaded to the Gene expression Omnibus^81^ under the SuperSeries accession number GSE178304 (microglia bulk RNA-seq in GSE178296; 6-month-old snRNA-seq data in GSE208683; 3-month-old snRNA-seq data in GSE178295). External datasets recruited for analysis were accessed through accession numbers listed in the Methods. Source data are provided with this paper.

## Code availability

Packages involved in data analysis pipelines are listed in the Methods. The code used for bulk and single-nuclei transcriptome sequencing is available on GitHub at https://github.com/TSun-tech/AD_MyelinMutant.git. The Fiji macro script for analysis of plaque-corralling microglia has also been deposited into GitHub at https://github.com/TSun-tech/AD_MyelinMutant.git. More detailed information is available upon contacting the corresponding authors.
