# Supplementary material for: Myelin dysfunction drives amyloid-β deposition in models of Alzheimer’s disease
Source: Nature. 2023 May 31;618(7964):349–57. doi: 10.1038/s41586-023-06120-6 (PMC10247380; doi:10.1038/s41586-023-06120-6)

nature portfolio

https://doi.org/10.1038/s41586-023-06120-6

Supplementary information

Myelin dysfunction drives amyloid-β deposition in models of Alzheimer's disease

In the format provided by the authors and unedited

Supplementary Information Figure Guide

SI Figure 1. Source data for western blot analysis
SI Figure 2. Myelination and gliosis in myelin mutant and aged mice (SI for Extended Data Figures 1d)
SI Figure 3. Assessment of *in toto* amyloid burden by light sheet microscopy

Supplementary Table 1.
Tab 1. Bulk RNA-seq DESeq2 analysis result summary
Tab 2. snRNA-seq individual sample preprocessing parameters

Tab 3. Microglia subsets analysis parameters
Tab 4. 3-month-old snRNA-seq microglia subpopulation marker gene list

Tab 5. 6-month-old snRNA-seq microglia subpopulation marker gene list

Tab 6. 6-month-old snRNA-seq microglia subset Myelin DAM vs Amyloid DAM DEGs
Tab 7. 6-month-old snRNA-seq microglia subset within DAM subpopulations DEG analysis
Tab 8. Bulk RNA-seq DEG (adjP<0.01) expression in TPM and corresponding kmeans cluster

Supplementary Table 2.
Tab 1. GSEA biological process enrichment analysis for DEGs between Cnp$^{-/-}$ 5xFAD vs 5xFAD
Tab 2. Gprofiler biological process enrichment analysis for DEGs between Cnp$^{-/-}$ 5xFAD vs 5xFAD
Tab 3. GSEA biological pathways enrichment analysis for DEGs between Cnp$^{-/-}$ 5xFAD vs 5xFAD
Tab 4. Gprofiler biological pathways enrichment analysis for DEGs between Cnp$^{-/-}$ 5xFAD vs 5xFAD
Tab 5-14. Gprofiler biological process enrichment analysis for DEGs in clusters 1 to 10.

Supplementary Information Figure legends

SI Figure 1. Source data for western blot analysis
(a-j) Uncropped Western blot raw data. Molecular weight marker is indicated

on the left (given in kDa). Red boxes indicate cropped regions as presented in the figures and the extended data figures. For normalisation, fast green total protein staining was performed on the same blot. Corresponding figure /extended data figure is indicated above each blot.

SI Figure 2. Myelination and gliosis in myelin mutant and aged mice (SI for Extended Data Figures 1d).
(a) Raw data shown as heatmap in Extended Data Figure 1 d. The percentage a rea positive for immunostaining (MBP for myelin profiles, GFAP for astrogli osis and IBA1 for microgliosis) was quantified in the indicated ROIs. m: mo nths. Bars represent means; dots represent individual mice/biological repli cate/n (n=3 per group). Statistical analysis: ordinary one-way ANOVA (p-val ue is given in graphs).
(b) Results of Tukey' s posthoc test for all comparisons after ordinary one -way ANOVA on data shown in (a).

SI Figure 3. Assessment of *in toto* amyloid burden by light sheet microscopy. **(I)** Brains were subjected to *in toto* staining with the β-sheet dye Cong o Red according to a modified iDisco protocol (see Material and Methods) fo llowed by clearing in Ethylcinnamate (Eci).
(II) Cleared brains were imaged on an Ultramicroscope II (LaVision-Biotech) light sheet setup to obtain sagittal optical slices.
(III) Raw data were visualised and analysed in Arivis Vision 4D using manua l region of interest annotation for hippocampus and cortex and automated pl aque segmentation (intensity-thresholding: 3-month-old *5xFAD*, blobfinder al gorithm: 6-month-old *5xFAD*, machine learning: 6-month-old *App*^{NLGF}).

Supplementary Figure 1

a Western Blot uncropped Main Figure 3f

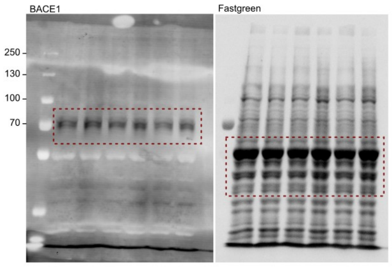

b Western Blot uncropped Extended Data Figure 6l

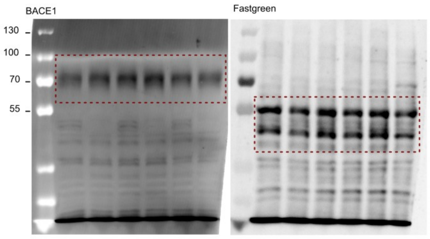

c Western Blot uncropped Main Figure 3g and Extended Data Figure 6m

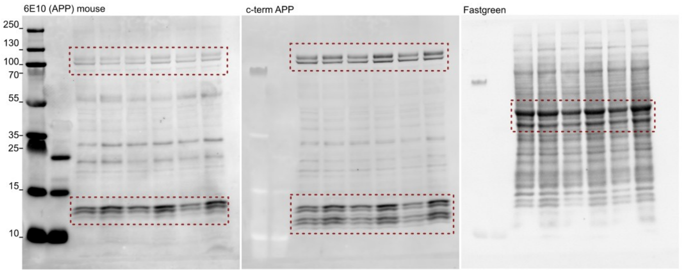

d Western Blot uncropped Extended Data Figure 6n

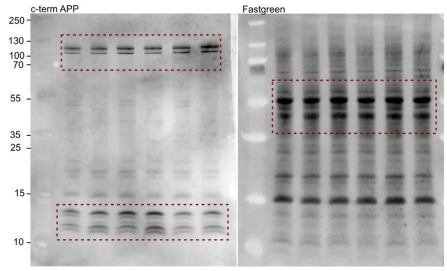

e Western Blot uncropped Extended Data Figure 10a

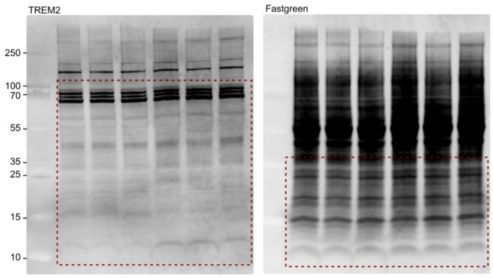

f Western Blot uncropped Extended Data Figure 10a

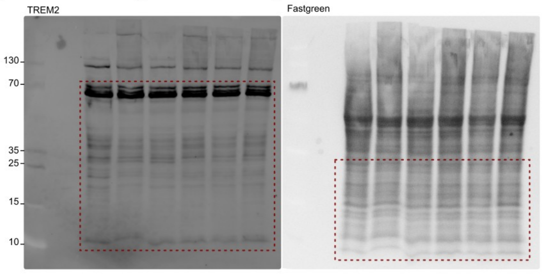

g Western Blot uncropped Extended Data Figure 10g

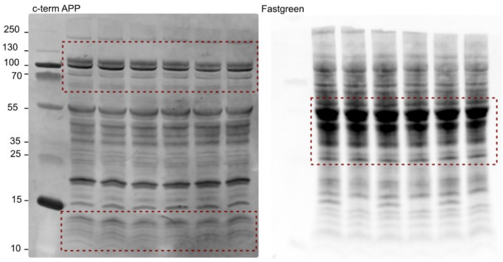

h Western Blot uncropped for myelin proteins in whole hemispheres of WT, 5xFAD, and NLGF mice (Extended Data Figure 3d)

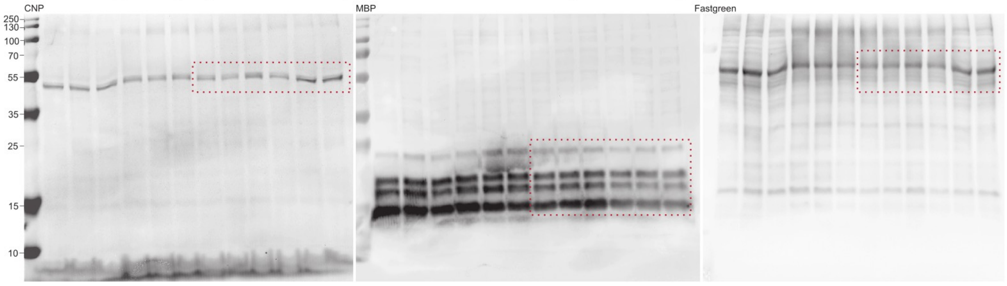

i Western Blot uncropped for myelin proteins in cortical fractions of WT, 5xFAD, and NLGF mice (Extended Data Figure 3d)

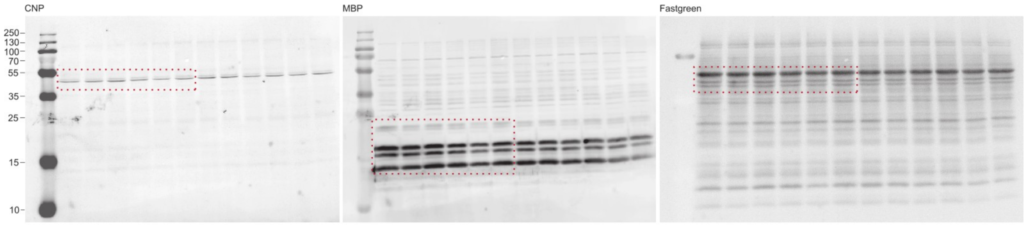

j Western Blot uncropped for myelin proteins in hippocampal fractions of WT, 5xFAD, and NLGF mice (Extended Data Figure 3d)

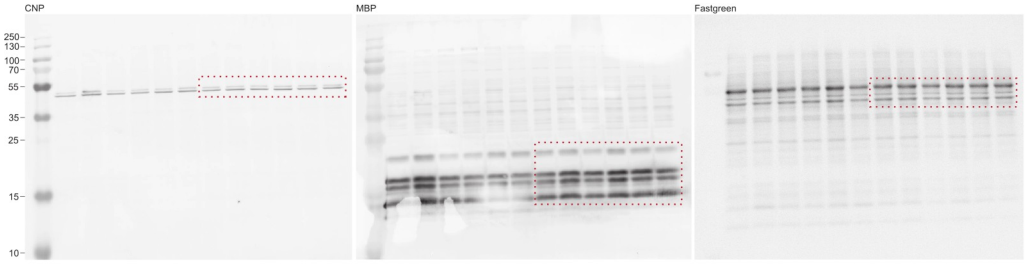

a Quantification of gliosis and myelin contant in myelin mutants

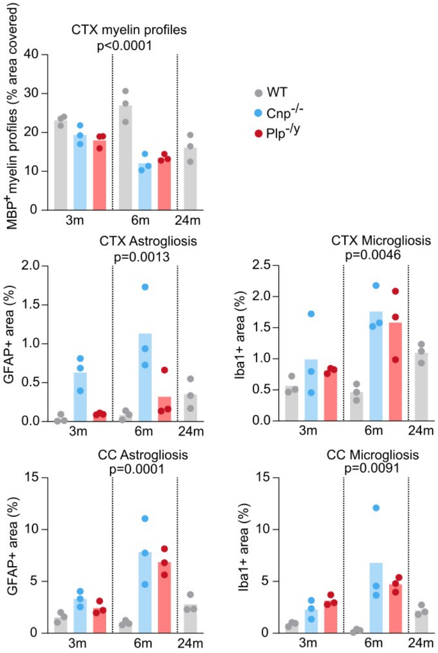

b Statistical analysis

	CTX			CC	
Comparisons	Myelin	GFAP	Iba1	GFAP	Iba1
WT 6m vs. $Cnp^{-/-}$ 6m	<0.0001	0.0027	0.0092	0.0005	0.0087
WT 6m vs. $Plp^{-/y}$ 6m	0.0002	0.9075	0.0274	0.0021	0.1031
WT 6m vs. WT 24m	0.0016	0.8504	0.3925	0.6962	0.8357
WT 6m vs. WT 3m	0.5245	>0,9999	0.9998	0.9986	0.9994
WT 6m vs. $Cnp^{-/-}$ 3m	0.0291	0.1898	0.5856	0.4384	0.8052
WT 6m vs. $Plp^{-/y}$ 3m	0.0083	>0,9999	0.8965	0.8561	0.4881
$Cnp^{-/-}$ 6m vs. $Plp^{-/y}$ 6m	0.9903	0.0211	0.9959	0.9728	0.8021
$Cnp^{-/-}$ 6m vs. WT 24m	0.4646	0.0275	0.3413	0.0077	0.0914
$Cnp^{-/-}$ 6m vs. WT 3m	0.0013	0.0018	0.0171	0.0011	0.0188
$Cnp^{-/-}$ 6m vs. $Cnp^{-/-}$ 3m	0.0369	0.2675	0.2054	0.0175	0.102
$Cnp^{-/-}$ 6m vs. $Plp^{-/y}$ 3m	0.1227	0.003	0.0753	0.0044	0.2516
$Plp^{-/y}$ 6m vs. WT 24m	0.8497	>0,9999	0.6681	0.0368	0.6343
$Plp^{-/y}$ 6m vs. WT 3m	0.0047	0.8163	0.0504	0.0051	0.2057
$Plp^{-/y}$ 6m vs. $Cnp^{-/-}$ 3m	0.1255	0.7439	0.467	0.0818	0.6716
$Plp^{-/y}$ 6m vs. $Plp^{-/y}$ 3m	0.3564	0.928	0.2024	0.0208	0.9303
WT 24m vs. WT 3m	0.0476	0.7404	0.579	0.9203	0.9687
WT 24m vs. $Cnp^{-/-}$ 3m	0.6839	0.8193	0.9998	0.9991	>0,9999
WT 24m vs. $Plp^{-/y}$ 3m	0.9642	0.8776	0.9557	0.9999	0.9946
WT 3m vs. $Cnp^{-/-}$ 3m	0.5447	0.1329	0.777	0.7164	0.9566
WT 3m vs. $Plp^{-/y}$ 3m	0.2205	>0,9999	0.978	0.9835	0.7322
$Cnp^{-/-}$ 3m vs. $Plp^{-/y}$ 3m	0.9912	0.2101	0.9955	0.9849	0.997

Protocol *in toto* amyloid plaque burden

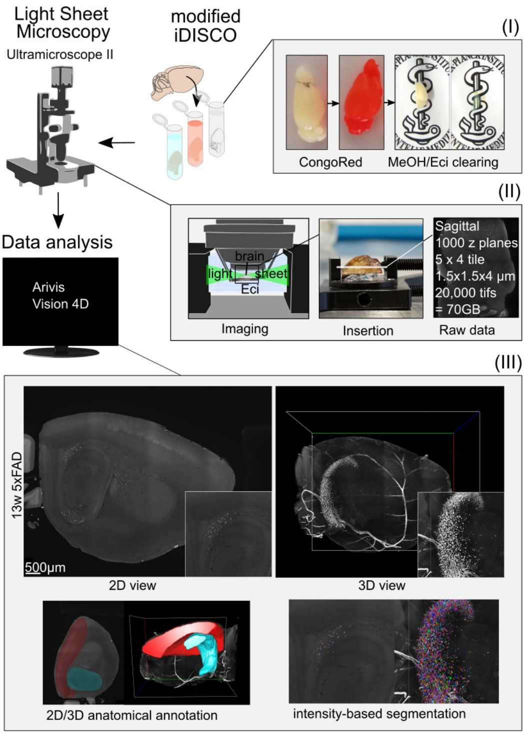

Supplement: Supplementary file 1 — Supplementary Guide and Supplementary Figs. 1–3. [file 41586_2023_6120_MOESM1_ESM.pdf]
