# Supplementary material for: Myelin dysfunction drives amyloid-β deposition in models of Alzheimer’s disease
Source: Nature. 2023 May 31;618(7964):349–57. doi: 10.1038/s41586-023-06120-6 (PMC10247380; doi:10.1038/s41586-023-06120-6)
Supplement: Supplementary file 2 — Reporting Summary [file 41586_2023_6120_MOESM2_ESM.pdf]

nature portfolio

Corresponding author(s): Klaus-Armin Nave
Constanze Depp

Last updated by author(s): March 2023

Reporting Summary

Nature Portfolio wishes to improve the reproducibility of the work that we publish. This form provides structure for consistency and transparency in reporting. For further information on Nature Portfolio policies, see our Editorial Policies and the Editorial Policy Checklist.

Statistics

For all statistical analyses, confirm that the following items are present in the figure legend, table legend, main text, or Methods section.

n/a	Confirmed	
☐	☒	The exact sample size (n) for each experimental group/condition, given as a discrete number and unit of measurement
☐	☒	A statement on whether measurements were taken from distinct samples or whether the same sample was measured repeatedly
☐	☒	The statistical test(s) used AND whether they are one- or two-sided<br>*Only common tests should be described solely by name; describe more complex techniques in the Methods section.*
☒	☐	A description of all covariates tested
☐	☒	A description of any assumptions or corrections, such as tests of normality and adjustment for multiple comparisons
☐	☒	A full description of the statistical parameters including central tendency (e.g. means) or other basic estimates (e.g. regression coefficient) AND variation (e.g. standard deviation) or associated estimates of uncertainty (e.g. confidence intervals)
☐	☒	For null hypothesis testing, the test statistic (e.g. F, t, r) with confidence intervals, effect sizes, degrees of freedom and P value noted<br>*Give P values as exact values whenever suitable.*
☒	☐	For Bayesian analysis, information on the choice of priors and Markov chain Monte Carlo settings
☒	☐	For hierarchical and complex designs, identification of the appropriate level for tests and full reporting of outcomes
☐	☒	Estimates of effect sizes (e.g. Cohen's d, Pearson's r), indicating how they were calculated

Our web collection on statistics for biologists contains articles on many of the points above.

Software and code

Policy information about availability of computer code

Data collection	Imaging data were acquired using ZEN 2011 (v2.0, Zeiss) and ImspectorPro (v. 7_124, LaVision Biotech)
Data analysis	Statistical testing was performed in Prism8.0 (Graphpad) or R (version 4.04, R Core Team 2021), afex package 0.28-1.<br>For image analysis: Vision 4D (Arivis, Versions: 3.0, 3.1, 3.2), FIJI v1.53c (Open source)<br>For sequencing analysis: CellRanger toolkit v3.0.2 and CellRanger toolkit v6.1.2 (10x Genomics), R package Seurat v3.2.3., Seurat v4.1.1, STRING (v11.5), MAST v1.20.0, UMAP v0.5.3, simplifyEnrichment package v1.5.2, gprofiler2 package v0.2.0, DESeq2 v.1.26.0, STAR v2.5.2b-2, featureCounts v1.6.3, FASTQC v0.72<br>The code used for bulk and single-nuclei transcriptome sequencing as well as FIJI macro to analyse plaque-coralling is available on GitHub https://github.com/TSun-tech/AD_MyelinMutant.git

For manuscripts utilizing custom algorithms or software that are central to the research but not yet described in published literature, software must be made available to editors and reviewers. We strongly encourage code deposition in a community repository (e.g. GitHub). See the Nature Portfolio guidelines for submitting code & software for further information.

Data

Policy information about availability of data

All manuscripts must include a data availability statement. This statement should provide the following information, where applicable:

- Accession codes, unique identifiers, or web links for publicly available datasets
- A description of any restrictions on data availability
- For clinical datasets or third party data, please ensure that the statement adheres to our policy

All raw sequencing data, as well as raw and processed counts matrices have been uploaded to the Gene expression Omnibus (GEO)101 under the SuperSeries

accession number GSE178304 (microglia bulk RNA-seq in GSE178296; 6-month-old snRNA-seq data in GSE208683; 3-month-old snRNA-seq data in GSE178295). The Allen mouse brain atlas is publicaly available under https://mouse.brain-map.org/experiment/thumbnails/100042147?image_type=atlas
nRNA-seq data from Mathys et al. 2019 (snRNAseqPFC_BA10) and Zhou et al. 2020 (snRNAseqAD_TREM2) were obtained from AD Knowledge Portal (https://adknowledgeportal.org).

Field-specific reporting

Please select the one below that is the best fit for your research. If you are not sure, read the appropriate sections before making your selection.

☒ Life sciences ☐ Behavioural & social sciences ☐ Ecological, evolutionary & environmental sciences

For a reference copy of the document with all sections, see nature.com/documents/nr-reporting-summary-flat.pdf

Life sciences study design

All studies must disclose on these points even when the disclosure is negative.

Sample size	Precalculations of sufficient sample size was not possible as biological effect sizes of the various experimental interferences could not be predetermined. Sample size dependent on availability of mice and previous experiences with the respective technique.
Data exclusions	No data were excluded.
Replication	Unless stated otherwise, individual mice were seen as independent replicates. Each experiment was replicated at least 3 times (exact n-numbers (number of independent biological replicates for each experiment are given in respective figure legends). For single cell analysis, two animals were pooled per replicate and two replicates were analysed. For in vitro analysis of myelin/amyloid phagocytosis, individual coverslips were seen as replicates. The experiment was performed one time.
Randomization	In our study, most experimental cohorts were defined by genotype. In the case of demyelination experiments (cuprizone treatment and EAE), animals were randomly assigned to treatment or non-treatment groups.
Blinding	Experimentators were blinded to genotype while performing behavioural testing or image analysis. In some cases, genotype of the analysed animals can be inferred due to gross morphological changes in myelin (i.e. EAE, CNP knockout) or mice body weight. For biochemical analysis, investigators were not blinded to genotype (loading scheme had to be known). For sequencing analysis, experimentators were not blinded to genotype in order to correctly identify cell clusters (e.g. 5xFAD DAM and Myelin DAM). Blinding is not relevant here as analysis is performed in a unbiased fashion.

Reporting for specific materials, systems and methods

We require information from authors about some types of materials, experimental systems and methods used in many studies. Here, indicate whether each material, system or method listed is relevant to your study. If you are not sure if a list item applies to your research, read the appropriate section before selecting a response.

Materials & experimental systems

n/a	Involved in the study	
☐	☒	Antibodies
☒	☐	Eukaryotic cell lines
☒	☐	Palaeontology and archaeology
☐	☒	Animals and other organisms
☐	☒	Human research participants
☒	☐	Clinical data
☒	☐	Dual use research of concern

Methods

n/a	Involved in the study	
☒	☐	ChIP-seq
☒	☐	Flow cytometry
☒	☐	MRI-based neuroimaging

Antibodies

Antibodies used	For immunohistochemistry: anti-Iba1 (rabbit, Wako 019-19741; 1:1000); anti-Aß-6E10 (mouse, Biolegend 803001; 1:1000), anti-CNP (mouse, AMAb91072, Atlas; 1:1000), anti-PLP-clone aa3 (rat, culture supernatant; 1:200; custom), 1:500 anti-BACE1(rabbit, ab183612, Abcam; 1:100), anti-MBP (rabbit, serum, custom Nave Lab; 1:1000), mouse anti-GFAP (mouse, GA5, Leica, 1:200) , anti-n-terminal APP (22c11, Merck MAB348; 1:1000), anti-c-terminal APP (1:1000, rabbit, A8717, Merck), anti-c-terminal APP (rabbit, 127-003, Synaptic Systems; 1:1000), anti-APP/Aß-D3E10 (rabbit, Cell Signalling Technology mAb #12843; 1:1000), anti-ApoE D7I9N (rabbit, Cell Signalling Technology mAb #13366; 1:500), anti-ApoE clone 26c11 (mouse, 1:500, provided by Christian Haass Lab), sAPPβswe clone 6A1 (mouse, IBL 10321, 1:1000), PSEN2 (rabbit; 1:100; Abcam ab51249).<br>Secondary antibodies in IHC: anti-mouse Alexa555 (donkey/goat, Thermo-Fisher A-21422 and A-31570; 1:1000), anti-mouse Dylight633 (goat, Thermo-Fisher 35512; 1:1000), anti-rabbit Alexa555 (donkey/goat, Thermo-Fisher A-21428 A-31572; 1:1000), anti-rabbit Dylight633 (goat, Thermo-Fisher 35563; 1:1000), goat anti-mouse DyLight488 (Thermo Fisher 35502; 1:1000).<br>For immunoblotting: anti-BACE1(1:1000, rabbit, D10E5, Cell Signalling Technologies 5606S), anti-c-terminal APP (1:1000, rabbit, A8717, Merck), anti-c-terminal APP (rabbit, 127-003, Synaptic Systems; 1:1000), anti-APP/Aß (1:1000, mouse, 6E10, Biolegend

	803001), anti-c-terminal TREM2 (1:1000, rabbit, E7P8J, Cell Signalling Technologies #76765)<br>Secondary antibodies for immunoblotting: anti-rabbit IgG (H+L) DyLight 800 (1:1000, Thermo Fisher SA5-10036), anti-mouse IgG (H+L) Dylight 680 (1:1000, Thermo Fisher # 35518).
Validation	Most of the antibodies used in this study are commercially available and have been validated by the manufacturers (see technical data sheets accessable on the manufacturers' websites). Where applicable, further validation was performed on 5xFAD and wildtype tissue. Staining or immunoblotting performance was evaluated based on comparison to typical staining patterns that should be observed in 5xFAD animals according to published studies (e.g. staining of amyloid plaques or corralling glia cells, upregulation in 5xFAD mice). This was performed for: anti-Iba1 (rabbit, Wako); anti-Aß-6E10 (mouse, Biolegend), anti-BACE1(rabbit, Abcam), anti-APP/Aß-D3E10 (rabbit, Cell Signalling Technology), mouse anti-GFAP (mouse, GA5, Leica), anti-n-terminal APP (22c11, Merck), anti-c-terminal APP (rabbit, A8717, Merck), anti-c-terminal APP (rabbit, 127-003, Synaptic Systems), anti-ApoE D7I9N (rabbit, Cell Signalling Technology), anti-c-terminal TREM2 rabbit, E7P8J, Cell Signalling Technologies), anti-BACE1(1:1000, rabbit, D10E5, Cell Signalling Technologies), PSEN2 (rabbit; Abcam ab51249). anti-ApoE D7I9N (rabbit, Cell Signalling Technology) and anti-ApoE clone 26c11 (mouse, 1:500, provided by Christian Haass Lab) were validated against Cuprizone-treated ApoE knockout animals.<br>Myelin protein specific antibodies (anti-MBP (rabbit, serum, custom Nave Lab; 1:1000), anti-PLP-clone aa3 (rat, culture supernatant), anti-CNP (mouse, Atlas) were validated in house against the corresponding knockout animal.

Animals and other organisms

Policy information about studies involving animals; ARRIVE guidelines recommended for reporting animal research

Laboratory animals	This study involved mice on the C57/B6 background. Transgenic animals used were: 5xFAD (Oakley et al., 2006) , APP NLGF (Saito et al., 2014), CNP-/- (Lappe-Siefke et al., 2004), PLP-/y (Griffiths et al., 1998), MBPfl/fl (Meschkat et al., 2020), Emx-Cre (Gorski et al., 2002), Foxg1-Cre(Kawaguchi et al., 2016), PLPfl/fl (Lueder et al., 2017). This study employed mice of various ages as indicated in the figures throughout the paper (3 to 24-month old). For CNP-/- crossbreedings, all analysed animals were females. For PLP-/y crossbreedings, all analysed animals were male. For Emx-Cre MBP fl/fl, both sexes were used. In cuprizone and EAE experiments, male animals were used. For Foxg1-Cre MBP fl/fl male mice were used. Mice were group-housed in the local animal facility of the Max Planck Institute for Multidisciplinary Sciences under a 12-h dark/12-h light cycle and fed ad-libitum (temperature of 22°C, 30–70% humidity).
Wild animals	Study did not involve wild animals.
Field-collected samples	Study did not include field-collected samples.
Ethics oversight	Animal experiments were overseen by the animal welfare officer and veterinarians of the Max-Planck-Institute for Experimental Medicine and where applicable approved by the local authorities (Landesamt für Verbraucherschutz und Lebensmittelsicherheit, Niedersachsen).

Note that full information on the approval of the study protocol must also be provided in the manuscript.

Human research participants

Policy information about studies involving human research participants

Population characteristics	Paraffin-embedded samples of selected patients (see recruitment) were anonymized and processed in a blinded manner. Selected patients were of mixed age (between >60 years and <90years of age) and gender had a clinical history of dementia, and a NIA-AA score in neuropathological assessment between A2B3C2 and A3B2C2 (inclusion criteria) and did not suffer from another severe neurological disorder (exclusion criteria). In addition, control patients of the same age range without any clinical or neuropathological record of neurological disease were selected. No other criteria besides the described characteristics were applied. In total 3 patients with moderate to pronounced AD neuropathological changes according to the NIA-AA (see above) and 3 control patients were histologically evaluated.
Recruitment	Case selection was performed from a pool of approximately 400 individuals, in which an autopsy with neuropathological evaluation was performed between 2018 and 2019 as a matter of routine procedure following death at the Leipzig University Hospital. Selection of patients was performed according to exclusion/inclusion criteria. Samples were anonymized and processed in a blinded manner. Selected patients were of mixed age, between >60 years and <90 years of age, had a clinical history of dementia, and a NIA-AA score in neuropathological assessment between A2B3C2 and A3B2C2 (inclusion criteria) and did not suffer from another severe neurological disorder (exclusion criteria). In addition, control patients of the same age range without any clinical or neuropathological record of neurological disease were selected. No other criteria besides the described characteristics were applied.
Ethics oversight	The ethics oversight was performed by the Ethics board of the University Hospital Leipzig.<br>In the individual contracts that govern medical treatment, all patients included into theis study provided upon admission written consent to the scientific use of tissue removed and stored after autopsy.

Note that full information on the approval of the study protocol must also be provided in the manuscript.
